# Ribosomal Protein S6 Phosphorylation Is Involved in Novelty-Induced Locomotion, Synaptic Plasticity and mRNA Translation

**DOI:** 10.3389/fnmol.2017.00419

**Published:** 2017-12-21

**Authors:** Emma Puighermanal, Anne Biever, Vincent Pascoli, Su Melser, Marine Pratlong, Laura Cutando, Stephanie Rialle, Dany Severac, Jihane Boubaker-Vitre, Oded Meyuhas, Giovanni Marsicano, Christian Lüscher, Emmanuel Valjent

**Affiliations:** ^1^IGF, CNRS, INSERM, University of Montpellier, Montpellier, France; ^2^Department of Basic Neurosciences, Medical Faculty, University of Geneva, Geneva, Switzerland; ^3^INSERM U1215, Université de Bordeaux, NeuroCentre Magendie, Bordeaux, France; ^4^Montpellier GenomiX, BioCampus Montpellier, CNRS, INSERM, University of Montpellier, Montpellier, France; ^5^Department of Biochemistry and Molecular Biology, IMRIC, The Hebrew University-Hadassah Medical School, Jerusalem, Israel; ^6^Clinic of Neurology, Department of Clinical Neurosciences, Geneva University Hospital, Geneva, Switzerland

**Keywords:** mRNA translation, striatum, LTP (long-term potentiation), ribosomal proteins, rpS6 phosphorylation

## Abstract

The phosphorylation of the ribosomal protein S6 (rpS6) is widely used to track neuronal activity. Although it is generally assumed that rpS6 phosphorylation has a stimulatory effect on global protein synthesis in neurons, its exact biological function remains unknown. By using a phospho-deficient rpS6 knockin mouse model, we directly tested the role of phospho-rpS6 in mRNA translation, plasticity and behavior. The analysis of multiple brain areas shows for the first time that, in neurons, phospho-rpS6 is dispensable for overall protein synthesis. Instead, we found that phospho-rpS6 controls the translation of a subset of mRNAs in a specific brain region, the nucleus accumbens (Acb), but not in the dorsal striatum. We further show that rpS6 phospho-mutant mice display altered long-term potentiation (LTP) in the Acb and enhanced novelty-induced locomotion. Collectively, our findings suggest a previously unappreciated role of phospho-rpS6 in the physiology of the Acb, through the translation of a selective subclass of mRNAs, rather than the regulation of general protein synthesis.

## Introduction

The ribosomal protein S6 (rpS6) is a crucial structural component of the 40S ribosomal subunit (Meyuhas, 2015). Localized at the interface between the small and the large ribosomal subunits (Nygard and Nilsson, 1990), rpS6 is the first ribosomal protein shown to undergo phosphorylation (Gressner and Wool, 1974). Five phosphorylation sites (S235, S236, S240, S244 and S247) have been identified on the carboxy-terminal domain (Krieg et al., 1988). While rpS6 phosphorylation can be triggered by several kinases including p70 S6 kinases (Krieg et al., 1988; Bandi et al., 1993; Meyuhas, 2008), p90 ribosomal S6 kinases (Roux et al., 2007) and protein kinase A (Moore et al., 2009; Valjent et al., 2011; Yano et al., 2014; Biever et al., 2015a), its dephosphorylation is carried out by a single phosphatase, protein phosphatase 1 (Biever et al., 2015a).

In the brain, rpS6 phosphorylation is evoked by a wide variety of pharmacological and physiological stimuli thus making it a widely-used marker to track changes in neuronal activity (Knight et al., 2012; Biever et al., 2015b). Although the exact role of these phosphorylation events remains largely unknown, early studies proposed that the phosphorylation of rpS6 could participate in the positive regulation of global translation and later studies on its regulatory role in translation of a subset of mRNAs, known as TOP mRNAs (reviewed in Ruvinsky and Meyuhas, 2006; Meyuhas and Kahan, 2015). Enhanced rpS6 phosphorylation is frequently associated with increased levels of TOP mRNA-encoded proteins in various models of synaptic plasticity in hippocampal, cortical, and striatal neurons (Kelleher et al., 2004; Klann and Dever, 2004; Tsokas et al., 2005, 2007; Antion et al., 2008a,b; Bowling et al., 2014) as well as in various mouse models of neurological and neurodevelopmental disorders which display an enhanced global protein synthesis (Biever et al., 2015b). However, recent observations indicate that these positive correlations are not systematic (Biever et al., 2015a) and prompt the need to re-evaluate the impact of rpS6 phosphorylation on protein synthesis in neurons.

Here, we used rpS6 knockin mice (rpS6^P−/−^), in which all five phosphorylatable sites (S235, S236, S240, S244 and S247) were mutated to alanine residues (Ruvinsky et al., 2005), to investigate directly the relationship between rpS6 phosphorylation and mRNA translation, plasticity and behavior. We first tested rpS6^P−/−^ mice in multiple behavioral tasks and the only phenotype we observed was an enhanced novelty-induced locomotion. For the following studies, we therefore decided to focus on the striatum, including the dorsal (DS) and ventral (nucleus accumbens, Acb) parts, due to its high involvement in locomotion and reactivity to rpS6 phosphorylation upon pharmacological stimuli (Biever et al., 2015b). Rather than an effect on global translation, we observed that rpS6 phosphorylation deficiency results in impaired translation of a subset of mitochondria-related mRNAs present in the Acb. We also found that rpS6 phospho-mutant mice display altered long-term potentiation (LTP) of excitatory synaptic transmission in the Acb, but not in the DS. These data indicate that rpS6 phosphorylation regulates a specific subset of mRNAs and associated processes in one brain area, rather than regulating global translation.

## Materials and Methods

### Animals

*Drd2a-eGFP* (C57Bl/6) mice were generated as described previously (Gong et al., 2003). rpS6^P−/−^ knockin (C57Bl/6/Sv129/ICR; Ruvinsky et al., 2005) mice were generated by crossing heterozygous rpS6^P+/–^ mice. Each transgenic mouse was compared with wild-type littermates of the same genetic background. Male and female were used for all experiments. Animals were housed under standardized conditions with a 12 h light/dark cycle, stable temperature (22 ± 1°C), controlled humidity (55 ± 10%) and food and water *ad libitum*. All experiments were in accordance with the guidelines of the French Agriculture and Forestry Ministry for handling animals and were approved by the local Ethic Committee (A 34-172-41).

### Tissue Collection

Mice were killed by cervical dislocation and the heads were immersed in liquid nitrogen for 4 s. The brains were then removed and the striatum, hippocampus, or frontal cortex were rapidly dissected on an ice-cooled disc. For the dorsal striatum (DS) and nucleus accumbens (Acb), the brains were sectioned. The Acb was isolated from a ~1-mm thick coronal section located between 1.94 mm and 0.86 mm anterior to bregma and the DS between 0.86 mm and 0.14 mm. Both structures were dissected on an aluminum block on ice.

### Western Blot Analysis

The brain regions of interest were rapidly dissected on an ice-cooled disc, sonicated in 300 μl 10% sodium dodecyl sulfate (SDS) and boiled at 100°C for 10 min. In each experiment, samples from all animal groups were processed in parallel to minimize inter-assay variations. Protein quantification and western blots were performed as described (Biever et al., 2015a). The antibodies used are summarized in Table 1. For quantitative purposes, the optical density values of active phospho-specific antibodies were normalized to the detection of nonphospho-specific antibodies or to β actin values in the same sample and expressed as a percentage of control mice.

**Table 1:** List of primary antibodies.

Antigen	Species	Dilution	Supplier/Catalog no./References)
pS2448-mTOR	Rabbit	1:1000 (WB)	Cell signaling technology (#2976)
mTOR	Rabbit	1:1000 (WB)	Cell signaling technology (#2971)
pT389-p70S6K	Rabbit	1:1000 (WB)	Cell signaling technology (#9234)
pT421/S424-p70S6K	Rabbit	1:1000 (WB)	Cell signaling technology (#9204)
p70S6K	Rabbit	1:1000 (WB)	Cell signaling technology (#9202)
pT37/46–4EBP1	Rabbit	1:500 (WB)	Cell signaling technology (#2855)
4EBP1	Rabbit	1:500 (WB)	Cell signaling technology (#9644)
pT202/Y204-ERK1/2	Rabbit	1:2000 (WB)	Cell signaling technology (#9101)
ERK1/2	Rabbit	1:2000 (WB)	Cell signaling technology (#4695)
pS51-eIF2α	Rabbit	1:1000 (WB)	Cell signaling technology (#9721)
eIF2α	Rabbit	1:1000 (WB)	Cell signaling technology (#5324)
pS1108-eIF4G	Rabbit	1:1000 (WB)	Cell signaling technology (#2441)
pT56-eEF2	Rabbit	1:1000 (WB)	Cell signaling technology (#2331)
pS209-eIF4E	Rabbit	1:1000 (WB)	Cell signaling technology (#9741)
eIF4E	Rabbit	1:1000 (WB)	Cell signaling technology (#9742)
rpS6	Mouse	1:1000 (WB)	Cell signaling technology (#2317)
eEF1A	Mouse	1:1000 (WB)	Millipore (#05-235)
β-actin	Mouse	1:40000 (WB)	Abcam (#ab6276)
VMAT2	Rabbit	1:1000 (WB)	Abcam (#ab81855)
MAO-A	Rabbit	1:1000 (WB)	Abcam (#ab126751)
PSD95	Mouse	1:3000 (WB)	Santa Cruz (#sc-32290)
Synaptophysin	Mouse	1:5000 (WB)	Sigma-Aldrich (#s-5768)
nNOS	Mouse	1:1000 (WB); 1:200 (IF)	Sigma-Aldrich (#N218)
TH	Mouse	1:5000 (WB); 1:1000 (IF)	Millipore (#mab318)
DAT	Rat	1:1000 (WB)	Millipore (#mab369)
NET	Rabbit	1:1000 (WB)	Millipore (#mab5066P)
ChAT	Rabbit	1:1000 (WB)	Millipore (#mab143)
ChAT	Goat	1:500 (IF)	Millipore (#mab144)
NeuN	Mouse	1:1000 (IF)	Millipore (#mab377)
Calretinin	Mouse	1:1000 (WB)	Swant (#6B3)
Parvalbumin	Rabbit	1:1000 (IF)	Swant (#PV25)
RibP	Human	1:1000 (IF)	Immunovision (HPO-0100)
D2R	Rabbit	1:1000 (WB); 1:1000 (IF)	Frontier Institute (#D2R-Rb-Af960)
D1R	Mouse	1:1000 (IF)	Corvol et al. (2007)
DARPP-32	Mouse	1:1000 (WB); 1:1000 (IF)	Ouimet et al. (1984)
Gαolf	Rabbit	1:1000 (WB)	Hervé et al. (2001)
Puromycin	Mouse	1:1000 (WB)	David et al. (2012)

### Immunofluorescence

Tissue preparation and immunofluorescence were performed as described (Biever et al., 2015a). Primary antibodies used are listed in Table 1. Goat Cy3-coupled anti-rabbit (1:500, Jackson ImmunoResearch Laboratories), donkey Cy3-coupled anti-goat (1:1000, Jackson ImmunoResearch Laboratories) and goat Alexa Fluor 488-coupled anti-mouse (1:500, Invitrogen) secondary antibodies were used. Three slices per mouse were used in all immunofluorescence analyses (*n* = 3–4 mice/staining).

### Whole Cell Recordings

Coronal 200–250 μm slices of mouse brain were prepared in cooled artificial cerebrospinal fluid (ACSF) containing (in mM): NaCl 119, KCl 2.5, MgCl 1.3, CaCl_2_ 2.5, Na_2_HPO_4_ 1.0, NaHCO_3_ 26.2 and glucose 11, bubbled with 95% O_2_ and 5% CO_2_. Slices were kept at 32–34°C in a recording chamber superfused with 2.5 ml/min ACSF. Visualized whole-cell patch-clamp recording techniques were used to measure synaptic responses of putative D1R and D2R-MSNs of the striatum, identified by the presence of the eGFP of BAC transgenic mice by using a fluorescent microscope (Olympus BX50WI, fluorescent light U-RFL-T). Whole cell recordings in voltage clamp mode. The holding potential was −70 mV, and the access resistance was monitored by a hyperpolarizing step of −14 mV with each sweep, every 10 s. Experiments were discarded if the access resistance varied by more than 20%. The internal solution contained (in mM) 140 k-gluconate, 5 KCl, 10 HEPES, 0.2 EGTA, 2 MgCl_2_, 4 Na_2_ATP, 0.3 Na_3_GTP, and 10 sodium creatine-phosphate. Currents were amplified (Multiclamp 700B, Axon Instruments), filtered at 5 kHz and digitized at 20 kHz (National Instruments Board PCI-MIO-16E4, Igor, WaveMetrics). The liquid junction potential was small (–3 mV), and therefore traces were not corrected. Experiments were carried out in the presence of picrotoxin (100 μM). Synaptic currents were evoked by stimuli (50–100 μs) at 0.1 Hz through bipolar stainless steel electrodes placed on the slice. The magnitude of HFS (100 pulses at 100 Hz repeated four times at 0.1 Hz paired with depolarization at 0 mV)-induced LTP was determined by comparing average EPSCs that were recorded 25–30 min after induction to EPSCs recorded immediately before induction. Paired-Pulse ratio (PPR) was determined with an inter-stimulation interval of 50 ms. Spontaneous EPSCs were recorded with the same conditions and frequency and amplitudes of these currents were then analyzed using the Mini Analysis software package (v.4.3, Synaptosoft). For recordings of neuronal firing as a function of current step injection, rheobase and resting membrane potential (RPM), the internal solution contained (in mM): potassium gluconate 130, MgCl_2_ 4, Na_2_ ATP 3.4, Na_3_ GTP 0.1, creatine phosphate 10, HEPES 5 and EGTA 1.1. No clamp was imposed and RPM should not vary by more than 10% otherwise cell was discarded. Firing was determined from a series of current injections (500 ms duration, 50 pA steps).

### Assessment of Overall Protein Synthesis

#### Puromycin Incorporation in Whole Striatal Lysates

Puromycin incorporation method was performed as described (Biever et al., 2015a). Briefly, the brain regions of interest were rapidly dissected and homogenized using 20 up-and-down strokes of a prechilled glass homogenizer with 800 μl of polysomal buffer containing 50 mM Tris pH = 7.8, 240 mM KCl, 10 mM MgCl_2_, 250 mM D-sucrose, 2% Triton X-100, 20 μl/ml emetine, 5 mM DTT, 100 U/ml RNasin (Promega) and protease inhibitor cocktail (Roche). Samples were centrifuged for 5 min at 16,100 × *g* at 4°C and supernatant was incubated with 100 μg/ml of puromycin for 10 min at 4°C and then boiled for 10 min at 100°C. In order to increase the puromycylation efficiency to levels obtained in live cells at 37°C, the translation elongation inhibitor emetine was included in the lysis buffer (David et al., 2013). Under these conditions of translational arrest, puromycin incorporates the nascent chain and labels a single polypeptide per ribosome. Similarly to the ribopuromycylation (David et al., 2012) or PUNCH-P (Aviner et al., 2014) methods, this procedure provides a snapshot of the translational state at a specific time-point and is not comparable to metabolic labeling or SUNSET (Schmidt et al., 2009). Protein concentrations were determined using BCA protein assay (Pierce, Rockford, IL, USA) and samples were stored at −20°C for further western blot analyses.

#### Polysome Profiling

The polysome profiling approach was performed as described (Biever et al., 2015a). Briefly, the brain regions of interest were rapidly dissected and homogenized using 30 up-and-down strokes of a prechilled glass homogenizer with 800 μl of polysomal buffer containing Tris 20 mM pH = 7.2, 130 mM KCl, 10 mM MgCl_2_, 0.05% Nonidet P-40, 100 μg/ml cycloheximide, 2.5 mM DTT, 200 U/ml RNasin (Promega) and protease inhibitor cocktail (Roche) for 15 min on ice. Samples were centrifuged for 5 min at 16,100 × *g* at 4°C and supernatant containing 200 μg of RNA (for striatum, hippocampus and frontal cortex) or 100 μg of RNA (for DS and Acb) were loaded onto 10%–50% sucrose density gradients (50 mM Tris pH = 7.5, 25 mM KCl, 10 mM MgCl_2_, 1 mM DTT). Samples were centrifuged in a SW41Ti swing out rotor (Beckman Coulter) for 3 h at 36,000 rpm at 4°C. Ribosome profiling was performed using a density gradient fractionation system (Brandel) with upward displacement and continuous monitoring at 254 nm using a UA-6 detector.

### Assessment of Specific mRNA Translation

#### RNAseq and qRT-PCR Following Polysome Profiling

The DS or Acb of two mice were pooled and processed as described in the previous section. Per sample, 50 μl were kept as input (referred to as “input RNA”) to determine cytosolic steady-state mRNA levels and 100 μg of RNA were loaded onto 10%–50% sucrose gradients. Gradients were then fractionated and RNA of fractions containing ≥4 ribosomes (referred to as “heavy polysomal fractions”) was extracted using TRIZOL protocol according to the manufacturer’s instructions. The carrier glycoblue was added before RNA precipitation step during the TRIZOL protocol. To remove potential DNA contamination, fractions were treated with DNase-free kit treatment and removal (Invitrogen) according to the manufacturer’s instruction. After the RNA extraction procedure, heavy polysomal fractions of each sample were pooled.

#### RNAseq

Three biological replicates, each one composed of heavy polysomal fractions of two samples were analyzed by RNAseq (*n* = 4 mice per biological replicate). RNAseq libraries were constructed with the Truseq stranded mRNA sample preparation (Low throughput protocol) kit from Illumina. One-hundred nanogram of total RNA was used for the construction of the libraries. The first step in the workflow involves purifying the poly-A containing mRNA molecules using poly-T oligo attached magnetic beads. Following purification, the mRNA was fragmented into small pieces using divalent cations under elevated temperature. The cleaved RNA fragments were copied into first strand cDNA using SuperScript II reverse transcriptase, Actinomycin D and random hexamer primers. The second strand cDNA was synthesized by replacing dTTP with dUTP. These cDNA fragments were adenylated and ligated to adapters. The products were purified and enriched with 15 cycles of PCR. The final cDNA libraries were validated with a Fragment Analyzer system (Advanced Analytical Technologies, Ankeny, IA, USA) and quantified with a KAPA qPCR kit. For each sequencing lane of a flowcell V3, libraries were pooled in equal proportions, denatured with NaOH and diluted to 8 pM before clustering. Cluster formation, primer hybridization and single end-read 50 cycles sequencing were performed on cBot and HiSeq2000 (Illumina, San Diego, CA, USA), respectively. Due to contamination by *E. coli* sequences, multiple sequencing runs have been done to obtain a minimum of 14 million mouse sequences per sample passing the Illumina purity filter.

#### Bioinformatic RNAseq Analysis

Image analyses and base calling were performed using the Illumina HiSeq Control Software and the Real-Time Analysis component. Demultiplexing was performed using Illumina’s conversion software (bcl2fastq 2.17). The quality of the raw data was assessed using FastQC from the Babraham Institute and the Illumina software SAV (Sequencing Analysis Viewer). A splice junction mapper, TopHat 2.0.13 (Kim et al., 2013; using Bowtie 2.2.3 (Langmead and Salzberg, 2012)), was used to align RNAseq reads to the mouse genome (UCSC mm10) with a set of gene model annotations (genes.gtf downloaded from UCSC on May 23, 2014). Final read alignments having more than three mismatches were discarded. Then, the counting was performed with HTSeq count 0.6.1p1 (union mode; Anders et al., 2015). The data is from a strand-specific assay, the read has to be mapped to the opposite strand of the gene. Before statistical analysis, genes with less than 15 reads (cumulating all the analyzed samples) were filtered and thus removed. Differentially expressed genes were identified using the Bioconductor (Gentleman et al., 2004) package DESeq2 1.4.5 (Anders and Huber, 2010). Translational efficiency was estimated, for each gene and genotype, as the ratio between heavy polysomal fractions associated mRNA counts and cytosolic steady-state mRNA counts. The Xtail 1.1.5 (Xiao et al., 2016) R package was used to identify genes showing differential translation efficiency values between genotypes. Data were normalized using the Bioconductor (Gentleman et al., 2004) package DESeq2 1.4.5 (Anders and Huber, 2010) normalization method. Genes with adjusted *P* value less than 5% (according to the FDR method from Benjamini-Hochberg) were declared differentially expressed/differentially translated. To perform the functional analysis of the resulting list of genes with the Gene Ontology (GO) annotations, the topGO (Alexa et al., 2006) package from Bioconductor was used. Overrepresented GO terms were identified using Fisher’s exact test with the weight method that is implemented in the topGO package. As confidence threshold we used a *P* value of 1%. To perform this analysis the differentially expressed genes were compared with those of all known genes present in the annotation. The GO categories were found in the Org.Mm.e.g.db package (Carlson, 2017) based on the gene reporter EntrezGeneID.

#### cDNA Synthesis and Quantitative Real-Time PCR

Synthesis of cDNA was performed on total RNA (input fraction) and heavy polysomal RNA, which were reverse transcribed to first strand cDNA using the SuperScript^®^ VILO™ cDNA synthesis kit (Invitrogen). Resulting cDNA was used for quantitative real-time PCR (qRT-PCR), using 2× SYBR Green Mix and LC480 Real-Time PCR System (Roche) as described (Puighermanal et al., 2017). Analysis was performed using LightCycler^®^ 480 Software (Roche). Results are presented as linearized Cp-values normalized to housekeeping genes β-actin or Hprt2 and the ∆CP method was used to give the fold change. The primer sequences are indicated in Table 2.

**Table 2:** Sequences of PCR primers.

Genes	PCR primers	
	Forward	Reverse
*Cox7a2*	GCCCTTCGTCAGATTGCCCAG	TCAGATGCCCCGCCTTTCAGA
*Cox7c*	GTTTGCCGCACCTTTCTTTA	CAGAGACGAGGCATTGAATCT
*Slirp*	GGCCATGTAAGAAGGTGCACTGT	ACCCAACCCATGCCTCTGTGA
*Uqcrh*	GCGTGTCTTCCCGGTCACAG	TGACAAGGCTGAGGAGGACGA
*Uqcrb*	AGAGAGCCCTGGACCTGACT	TGCCCACTCTTCTCTCTCCTT
*Timm8a1*	TGCAGCATTTCATCGAGGTG	GACTGTCCAACTTAGGCCCA
*Mrpl33*	CTCCGAGAGAAGCTGAGCCT	CCACTACTCAGAGGGAACGGA
*Dbi*	CAAGTGGGACTCGTGGAACA	AGCTCGTCTACCTTTTCCACA
*β-actin*	CGTGAAAAGATGACCCAGATCA	CACAGCCTGGATGGCTACGT
*Hprt2*	GCAGTACAGCCCCAAAATGG	GGTCCTTTTCACCAGCAAGCT

#### Data Availability

Sequence data have been deposited in the Gene Expression Omnibus database (accession no. GSE98029).

### ATP Determination

The Acb of rpS6^P−/−^ mice and wild-type littermates were homogenized in Krebs-Ringers bicarbonate buffer (125 mM NaCl, 1.4 mM KCl, 20 mM HEPES [pH 7.4], 5 mM NaHCO_3_, 1.2 mM MgSO_4_, 1.2 mM KH_2_PO_4_, 1 mM CaCl_2_) containing 1% BSA. Protein concentrations were determined using the BCA assay (Pierce). To measure ATP concentration, 100 μg of Acb lysates were used for the CellTiter-Glo Luminiscent Cell Viability kit (Promega) in a 96-well format following manufacturer’s instructions.

### Mitochondria Assays

Complex I (EC 1.6.5.3) was measured by recording the decrease in absorbance due to oxidation of NADH (Roche) at 340 nm (*ε* = 6.2 mM^−1^ cm^−1^) in a POLARstar Omega plate reader (BMG Labtech) at 30°C in a volume of 1 ml. The Acb of rpS6^P−/−^ and wild-type littermates was dissected and stored at −80°C until further use. Tissue was homogenized with a glass potter in 150 μL of ice-cold Mir05 buffer (Mitochondrial Physiology Network: 0.5 mM EGTA, 3 mM MgCl_2_, 60 mM lactobionate, 20 mM taurine, 10 mM KH_2_PO_4_, 20 mM HEPES, 110 mM sucrose and 1 g L−1 BSA). The homogenate was centrifuged at 500× *g* for 10 min at 4°C to remove debris. The supernatant was kept on ice for the assays. Tissue homogenate (200–250 μg protein) was added to the assay buffer (35 mM K_2_HPO_4_ pH = 7.2, 5 mM MgCl_2_, 2.5 g L−1 BSA, 0.1 mM NADH) and the reaction was started by addition of 0.1 mM Decylubiquinone with changes in 340 nm absorbance (slope) measured for 3 min. Rotenone (12.5 μM) was added and monitoring carried for further 3 min. Complex I activity was taken as the rotenone-sensitive activity measured by the percent inhibition after addition of rotenone.

Citrate synthase (CS; E.C. 2.3.3.1) was measured by recording the increase in absorbance at 412 nm of the reaction of acetyl-CoA with Oxaloacetate (OAA) and the release of free CoA-SH to the colorimetric reagent 5,5-dithiobis(2-nitrobenzoate; DTNB; *ε* = 13.6 mM^−1^ cm^−1^) in a POLARstar Omega plate reader (BMG Labtech) at 37°C in a volume of 1 mL. Tissue homogenate (50–70 μg protein) was added to the assay buffer (20 mM Tris-HCl pH = 8, 0.1 mM DTNB, 0.1% Triton X-100) and the reaction was started by addition of 0.3 mM Acetyl-CoA, and 0.5 mM OAA with changes in 412 nm absorbance (slope) measured for 3 min. Assay conditions were controlled by using standard commercial CS (Sigma C3260) diluted 1:500 in PBS, pH = 7.0. Assay measurements were performed in duplicate. Brain samples were prepared as described above for Complex I.

### Dendritic Spine Morphology Analysis

Dendritic spine analysis was performed as previously described (Guegan et al., 2013). Briefly, rpS6^P−/−^ knockin mice and wild-type littermates were deeply anesthetized with pentobarbital (500 mg/kg, i.p.; Sanofi-Aventis) prior to rapid intracardiac perfusion, delivered with a peristaltic pump at 20 ml/min, with 10 ml of Na_2_HPO_4_/NaH_2_PO_4_/NaCl buffer (PBS) 0.1 M, pH 7.5, and followed by perfusion with 40 ml of 4% PFA in PBS 0.1 M, pH 7.5. Brains were postfixed in 4% PFA for 10 min and kept in PBS at 4°C. Brain coronal sections (100 μm) containing the Acb (Franklin and Paxinos, 2008; located between 1.54 mm and 0.98 mm anterior to Bregma) and DS (located between 1.42 mm and 0.14 mm anterior to Bregma) were obtained by using a vibratome (Leica) and stored at −20°C in a solution containing 30% (v/v) ethylene glycol, 30% (v/v) glycerol, and 0.1 M sodium phosphate buffer until they were processed for fluorescent labeling. Brain slices were labeled by ballistic delivery of fluorescent dye DiI (Molecular Probes, Eugene, OR, USA) using a gene gun apparatus (Helios Gene Gun System, Bio-Rad, Deutschland) and postfixed with PFA for 4 h at room temperature to further preserve structures and to allow the diffusion of the dye DiI. Sections were placed on microscope gelatine-coated slides and coverslipped with mounting medium. Acquisition and analysis of dendritic spine images was performed as described (Dumitriu et al., 2011, 2012). Z-Stacks were acquired using a 60 × 60 × 168 nm^3^ voxel size on an inverted Leica SP8 confocal laser scanning microscope at the Montpellier RIO Imaging Facility. Images were acquired with excitation at 561 nm and acquisition of the emission between 570 and 620 nm at a frequency of 400 Hz with a 63× 1.4 numerical aperture oil immersion objective. Images were averaged by four acquisitions to minimize noise. Laser intensity and photomultiplier gain were adjusted for each image to fill the entire range of the sensor and minimize the noise level. Secondary to tertiary dendrites of individual medium-sized spiny neurons were chosen for spine analysis Images were deconvolved with Huygens 3.5 (Scientific Volume Imaging) using an experimental Point Spread Function and spine analysis was performed using the semiautomated software NeuronStudio, which analyses dendritic length, dendritic width, spine number and spine head diameter in 3D. Parameters for dendritic spine quantification with NeuronStudio were: minimum neurite length = 10 μm; minimum spine height = 0.4 μm; maximum spine height = 3 μm; minimum stubby size = 20 voxels. Parameters for spine classification were: neck ratio = 1.1; thin ratio = 2.25; mushroom size = 0.4 μm. After NeuronStudio processing, verification of the dendritic spine densities quantification was performed in blind conditions and manually corrected if any errors in spine identification.

### Locomotor Activity

#### Open Field Test

Spontaneous exploratory behavior was monitored in an open field (white plastic box with 35 cm width × 45 cm length × 25 cm height) for 30 min. The open field was wiped with 70% ethanol between sessions. The center zone was defined as a virtual perimeter within 5 cm from the sides of the box. Experiments were filmed and an observer blinded to genotype scored the time spent in the center (4 paws inside the center zone) and the number of midline crosses (4 paws crossing a midline of the box) during the first 10 min. Videos were analyzed using Noldus EthoVision XT (Netherlands) software by calculating the total distance traveled, average speed, time spent immobile and immobility frequency.

#### Circular Corridor

Locomotor activity was measured during 20 min in a circular corridor with four infrared beams placed at 90° angles (Imetronic, Pessac, France) in a low luminosity environment. Counts for horizontal activity were incremented by consecutive interruption of two adjacent beams (mice moving through 1/4 of the circular corridor) and counts for vertical activity (rearings) corresponding to interruption of beams placed at a height of 7.5 cm along the corridor were used.

### Elevated Plus Maze

Anxiety-like behavior was measured as previously described (Busquets-Garcia et al., 2011).

### Marble Burying Test

Repetitive behaviors were assessed by marble burying activity. Mice were placed in a clean cage (46 × 20 × 14 cm) with 4 cm sawdust bedding overlayed by 20 glass marbles (15 mm diameter) equidistant in a 4 × 5 arrangement. Mice were allowed to explore the cage for 20 min and the number of marbles buried (>50% of the marble covered by the bedding) was counted.

### Tail Suspension Test

Mice were suspended 50 cm above a cushioned pad using tape to attach their tails to a horizontal pole above the pad. Each mouse was tested for a 6-min trial. Latency to the first bout of immobility (defined as ≥ 5 s-long segment of time spent immobile) and the total time spent immobile during the trial were recorded. Immobility was defined as hanging passively without any movement of the head or paws.

### Spontaneous Alternation Test

Spontaneous alternation was measured in a Plexiglas Y-shaped maze with three identical arms (40 × 9 × 16 cm) at a 120° angle. Each individual mouse was placed in the center of the maze and allowed to freely explore for 5 min. A triad was defined as a set of three arm entries, when each entry was to a different arm of the maze. The number of arm entries and the number of triads were recorded. The percentage of alternation was calculated by dividing the number of triads by the number of possible alternations and then multiplying by 100.

### Three-Chamber Social Approach

A three-chamber arena was used to assess sociability, preference for social novelty, and social memory. On day 1, stranger target mice were habituated to the wire cups. On day 2, test mice (2-month-old males) were placed in the middle chamber and allowed to explore all the empty chambers of the apparatus freely for 10 min. Next, an unfamiliar mouse (Stranger#1, male C57BL/6 age matched) was introduced into one of the two side chambers, enclosed in a wire cage allowing only for the test mouse to initiate any social interaction. An identical empty wire cage was placed in the other side chamber. Following placement, the test mouse was allowed to explore the whole three-chamber arena for 10 min. At the end of the 10 min sociability test, a new unfamiliar mouse (Stranger#2, male C57BL/6 age matched) was placed in the previously unoccupied wire cage, and test mice were examined for an additional 10 min to assess preference for social novelty. The time spent in each compartment and the time spent sniffing the Stranger#1, Stranger#2, or empty wire cages were manually scored.

### Statistical Analysis

All statistical analyses were performed using one-way analysis of variance (ANOVA) for multiple comparisons, followed by Newman-Keuls *post hoc* test. Student *t*-test with equal variances was used for groups of two, when relevant. Statistical significance was determined as *p* < 0.05. Prism 5.0 software was used to perform statistical analyses. Locomotor activity was analyzed by matched two-way ANOVA (effect of time and effect of genotype), followed by Bonferroni’s multiple comparison test. All statistical analyses are available in Supplementary Table S1.

## Results

### Absence of rpS6 Phosphorylation Enhances Novelty-Induced Locomotor Activity

**Figure 1:**
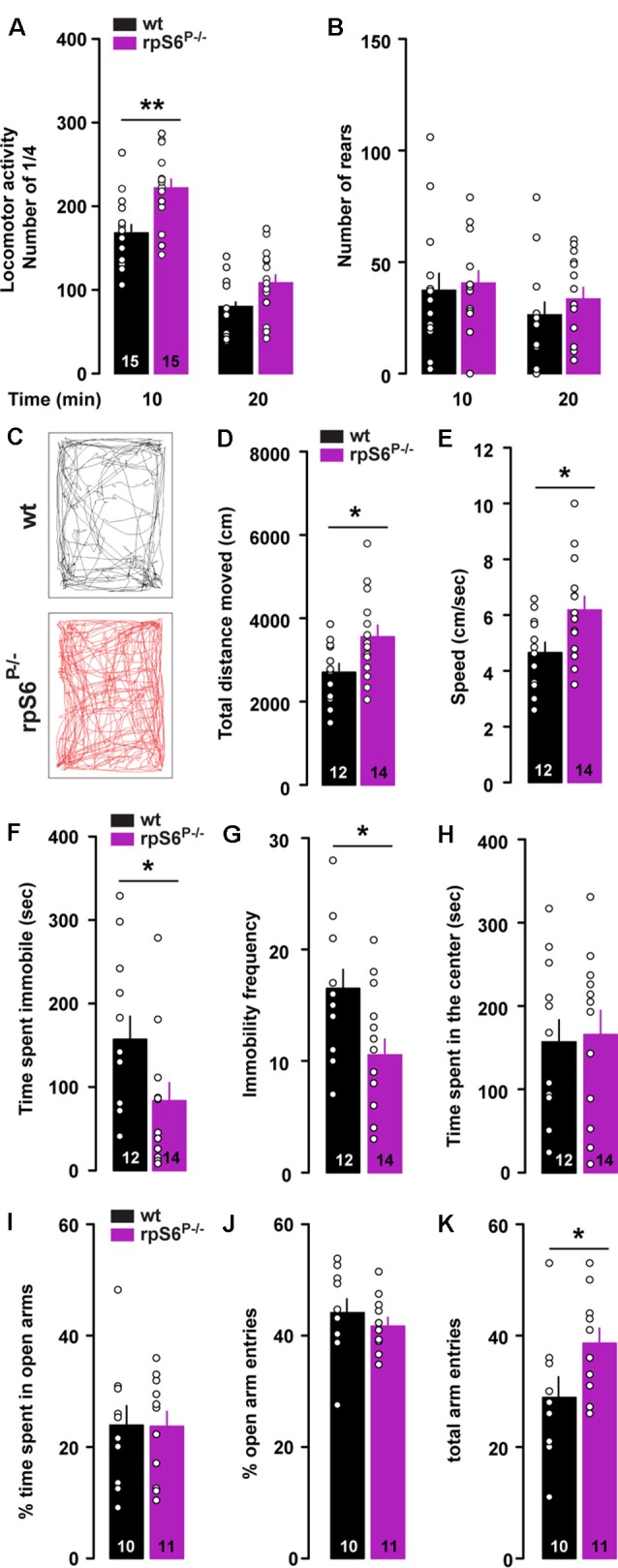
Enhanced novelty-induced locomotion in rpS6^P−/−^ mice. **(A)** Horizontal and **(B)** vertical (number of rears) locomotor activity in wild-type (wt, black) and rpS6 phospho-deficient mice (rpS6^P−/−^, magenta) measured in a circular corridor during 20 min. **(C)** Representative movement path of a wt (back) and rpS6^P−/−^ (red) mouse during the first 10 min in an open field. **(D)** Total distance moved (cm), **(E)** speed (cm/s), **(F)** time spent immobile (s) and **(G)** immobility frequency during the first 10 min. **(H)** Time spent in the center of the open area (s). **(I)** Percentage of time spent in the open arms of the elevated plus maze. **(J)** Percentage of open arms entries and **(K)** the number of total arm entries during 5 min. The number of animals in each condition is indicated in the bars. Results are represented as scatter plots and mean ± SEM. Statistical analysis, Student’s *t*-test (*n* and *p* values in Supplementary Table S1:1a,b,d–k), *p < 0.05; **p < 0.01.

To determine the physiological role of rpS6 phosphorylation, we used rpS6 knockin mice (rpS6^P−/−^), in which all phosphorylation sites were mutated to alanine residues (Supplementary Figure S1). We first monitored locomotor activity in non-stressful conditions (i.e., low luminosity) using circular corridors. A significant increase in horizontal locomotor activity was observed during the first 10 min in rpS6^P−/−^ mice (Figure 1A). In contrast, no differences were found in the number of rears (Figure 1B). This enhanced reactivity to a novel environment was further confirmed in mice video-tracked in an open field arena, as demonstrated by the significant increase in total distance traveled during the first 10 min in rpS6^P−/−^ mice (Figures 1C,D). This enhanced locomotor response was accompanied by an increased movement velocity (Figure 1E), a decrease in the total time spent immobile (Figure 1F) and the immobility frequency (Figure 1G). No difference in locomotor activity was found at longer time points (20 and 30 min) between genotypes in both the circular corridor and open field arena (Figure 1A and data not shown). The enhanced locomotion was not a result of novelty-induced anxiety since mice spent the same amount of time in the center of the open field (Figure 1H) and in the open arms in the elevated plus maze (Figures 1I,J). In the latter, the total arm entries were higher in rpS6^P−/−^ mice most likely due to the enhanced reactivity to novelty (Figure 1K). No differences between genotypes were found in other behavioral tasks including marble burying test, tail suspension test, Y-maze spontaneous alternation test and sociability as well as social novelty in the three-chamber test (Supplementary Figure S2). Together, our results indicate that novelty-induced locomotor activity is enhanced in rpS6^P−/−^ mice.

### Striatal Anatomy and Structural Plasticity Are Unaltered in rpS6^P−/−^ Mice

**Figure 2:**
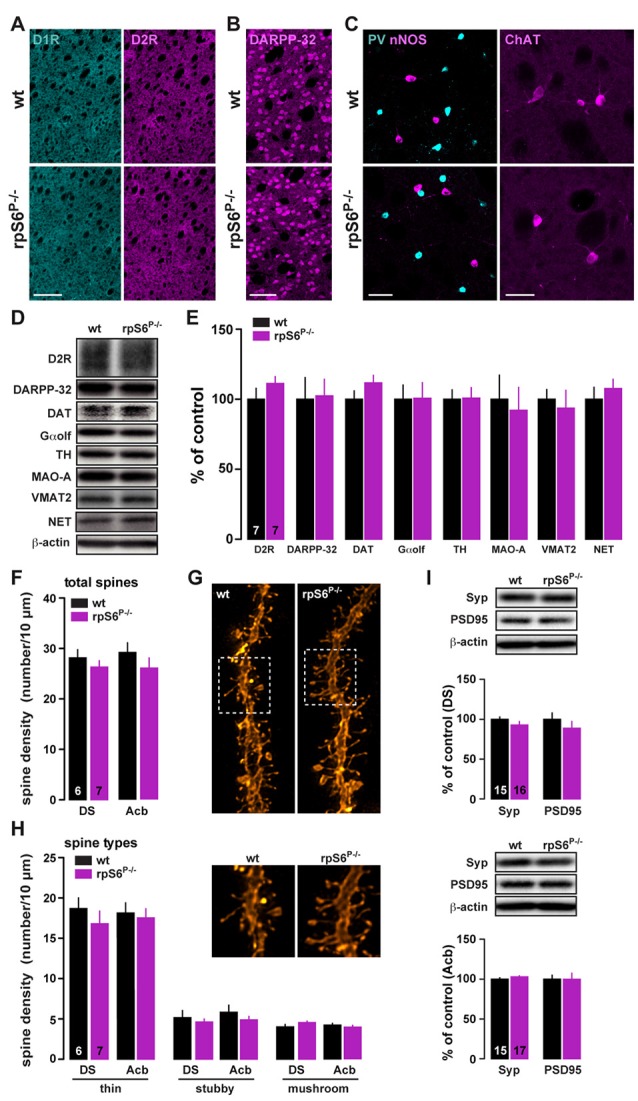
Striatal dendritic spines density and protein expression in rpS6^P−/−^ mice.** (A)** Single immunostaining for D1R (cyan) and D2R (magenta) in coronal sections of the striatum from wt (top) and rpS6^P−/−^ (bottom) mice. Scale bar: 250 μm. **(B)** Single immunostaining for DARPP-32 of the striatum from wt (top) and rpS6^P−/−^ (bottom) mice. Scale bar: 60 μm. **(C)** Double immunostaining for nNOS (magenta) and parvalbumin (PV, cyan; left panel) or single immunofluorescence for choline acetyltransferase (ChAT; right panel) in the striatum of wt (top) and rpS6^P−/−^ (bottom) mice. Scale bar: 40 μm and 20 μm. **(D)** Representative western blots and **(E)** quantified signals of D2R, DARPP-32, DAT, Gαolf, TH, MAO-A, VMAT2 and NET normalized to β-actin in whole striatal lysates from wt (black bars) and rpS6^P−/−^ (magenta bars) mice. Results are represented as mean ± SEM (*n* and *p* values in Supplementary Table S1:2e). **(F)** Total number of spines per 10 μm in DS and Acb MSNs of wt (black bars) and rpS6^P−/−^ (magenta bars) mice. **(G)** Representative dendrites spines visualized by fluorescent dye DiI. **(H)** Density of thin, stubby or mushroom dendritic spines were analyzed on a subset of dendrites in DS and Acb MSNs of wt (black bars) and rpS6^P−/−^ (magenta bars) mice. The number of animals in each condition is indicated in the bars. Results are represented as mean ± SEM (*n* and *p* values in Supplementary Table S1:2g,h). **(I)** Representative western blots and quantified signals of Syp and PSD95 normalized to β-actin in DS (TOP) and Acb (bottom) lysates from wt (black bars) and rpS6^P−/−^ (magenta bars) mice. Results are represented as mean ± SEM (*n* and *p* values in Supplementary Table S1:2i).

Altered size of several cell types has been reported in peripheral tissues of rpS6^P−/−^ mice (Ruvinsky et al., 2005, 2009). To determine whether rpS6 phosphorylation was also a critical determinant of neuronal size, we analyzed whether the brain was developed normally in adult rpS6 phosphorylation-deficient mice. No changes in the brain weight and size were observed between rpS6^P−/−^ mutant mice and wild-type littermates (Supplementary Figure S3). Histological analyses using the neuronal marker NeuN did not reveal any gross abnormality in cytoarchitecture in mutant mice (Supplementary Figure S3). No evident alterations of specific brain areas, including the DS and the Acb, were detected (Supplementary Figure S3). The expression levels of striatal proteins important for its functionality were also analyzed by immunofluorescence and western blots. No changes in dopamine D1 and D2 receptors expression were found (Figure 2A). The size, the distribution and the expression of medium-sized spiny neurons (DARPP-32) as well as different classes of striatal interneurons, including parvalbumin (PV), nNOS, calretinin (CR), and cholinergic (ChAT) interneurons were unaffected (Figures 2B,C and Supplementary Figure S4). Similarly, immunoblot analyses of striatal homogenates revealed no differences between genotypes in the expression levels of D2R, DARPP-32, dopamine transporter (DAT), guanine nucleotide-binding protein G(olf) subunit alpha (Gαolf), tyrosine hydroxylase (TH), monoamine oxidase A (MAO-A), vesicular monoamine transporter 2 (VMAT2) and norepinephrine transporter (NET) (Figures 2D,E).

To determine whether rpS6 phospho-deficiency could lead to discrete morphological changes, striatal slices of rpS6^P−/−^ and wild-type mice were labeled by ballistic delivery of fluorescent dye DiI. Analyses of dendritic spine density or types of spines (thin, stubby and mushroom) in labeled output neurons of the DS and Acb revealed no differences between genotypes (Figures 2F–H). In addition, equivalent contents of synaptophysin (Syp) and PSD95, pre- and post-synaptic markers respectively, were found (Figure 2I). Altogether, our results show that rpS6^P−/−^ mice display intact striatal morphology and normal levels of proteins involved in striatal function.

### Impaired HFS-Induced LTP in Both D1- and D2-MSNs in the Acb of rpS6^P−/−^ Mice

**Figure 3:**
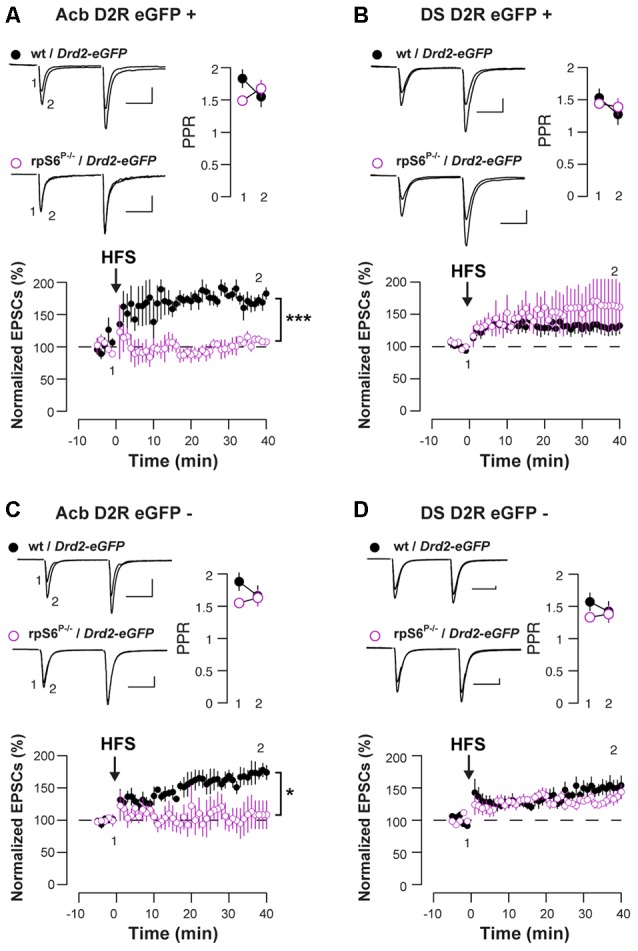
Altered HFS-induced long-term potentiation (LTP) in Acb D1-and D2-MSNs of rpS6^P−/−^ mice. EPSCs were recorded from eGFP positive (D2-MSNs) **(A,B)** and eGFP negative (D1-MSNs) **(C,D)** in the Acb **(A,C)** or in the DS **(B,D)** of wt/*Drd2*-eGFP (black circles) and rpS6^P−/−^/*Drd2*-eGFP (magenta open circles) double transgenic mice before and after HFS-induced LTP. Graphs show normalized EPSCs as a function of time and the overlay of averaged (20 trials) traces of AMPAR EPSCs before (1) and after (2) HFS. Scale bars: 50 pA, 20 ms. Symbols represent average of six trials. *p < 0.05; ***p < 0.001 by Student’s *t*-test (*n* and *p* values in Supplementary Table S1:3a–d).

Enhanced rpS6 phosphorylation is frequently observed in various electrical and chemical models of synaptic plasticity (Biever et al., 2015b). To distinguish between D1- and D2-MSNs, we crossed rpS6^P−/−^ mice with *Drd2*-eGFP mice and we recorded both eGFP-positive (D2-MSNs) and eGFP-negative cells (most likely D1-MSNs) in the Acb and DS. Baseline electrophysiological parameters were similar in rpS6^P−/−^ mice compared to control, with the exception of accumbal D2-MSNs that showed a slightly elevated threshold for action potential (AP) generation. However, in the absence of altered RMP and input-output curves, this likely reflects a sampling error. Taken together, passive membrane properties, AP generation, and overall excitatory synaptic inputs were not affected by the genetic manipulation (Supplementary Figure S5). We next examined synaptic transmission at excitatory afferents onto MSNs after a challenge with a high-frequency stimulation (HFS) train. While the HFS protocol induced a LTP of the excitatory postsynaptic currents (EPSCs) in D2-MSNs in the Acb of wild-type mice, it failed to do so in rpS6^P−/−^ mice (Figure 3A). This effect was specific of the Acb since no differences between genotypes were observed after recording D2-MSNs in the DS (Figure 3B). Interestingly, similar results were obtained when the analysis was performed in D1-MSNs (Figures 3C,D). Together, our results indicate that rpS6 phosphorylation deficiency predominantly impacts synaptic plasticity in the Acb.

### RpS6^P−/−^ Mice Display Normal Overall Protein Synthesis

**Figure 4:**
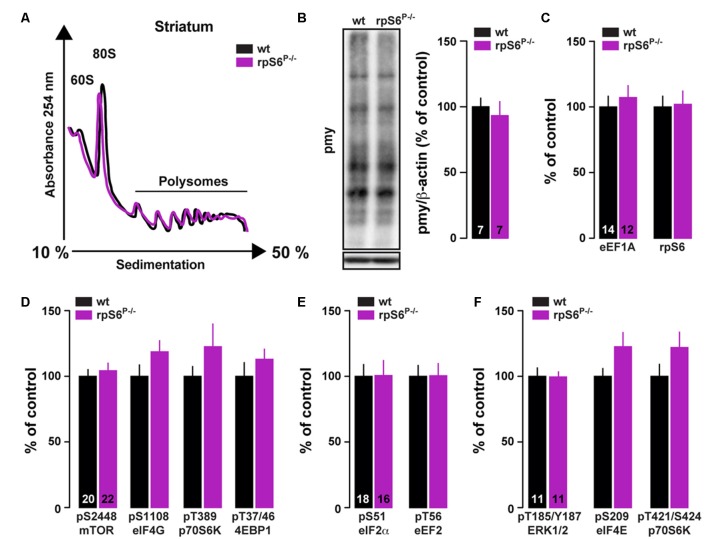
Analysis of global mRNA translation in the striatum of rpS6^P−/−^ mice.** (A)** Representative polysome profile of whole striatum lysates from wt (black line) and rpS6^P−/−^ (magenta line) mice (*n* = 6 mice/group). **(B)** Representative (left) and quantified (right) western blot analysis of puromycin staining (pmy, normalized to β-actin) in whole striatal lysates from wt (black bars) and rpS6^P−/−^ (magenta bars) mice incubated 10 min with puromycin. Results are represented as means ± SEM (*n* and *p* values in Supplementary Table S1:4b). **(C–F)** Quantified western blot analysis of eE1A and rpS6 **(C)**, pS2448-mTOR, pS1108-eIF4G, pT389-p70S6K and pT37/46-4EBP1 **(D)**, pS51-eIF2α and pT56-eEF2 **(E)**, pT185/Y187-ERK1/2, pS209-eIF4E and pT421/S424-pS70S6K **(F)** in total striatal lysates from wt (black bars) and rpS6^P−/−^ (magenta bars) mice. The number of animals in each condition is indicated in the bars. Results are represented as means ± SEM (*n* and *p* values in Supplementary Table S1:4c–f).

To investigate directly whether rpS6 phosphorylation participates in the fundamental control of mRNA translation in the nervous system, we performed polysome profile analysis on striatal lysates (comprising both the DS and the Acb) from rpS6^P−/−^ mice and wild-type littermates. As shown in Figure 4A, no difference in the polysome profiling was observed between genotypes. We also conducted polysome profiling analyses on frontal cortex or hippocampal lysates and failed to observe differences between genotypes (Supplementary Figure S6). Similar results were obtained when striatal *de novo* protein synthesis was assessed using an assay adapted from the ribopuromycylation method (David et al., 2012). Indeed, no difference in puromycin incorporation was found in striatal lysates of rpS6^P−/−^ mice compared wild-type littermates (Figure 4B). We next analyzed TOP-encoded proteins since increased rpS6 phosphorylation has been correlated with enhanced hippocampal TOP mRNA translation during LTP (Tsokas et al., 2005; Antion et al., 2008b). No changes in the TOP-encoded proteins rpS6 and eukaryotic translation elongation factor 1A (eEF1A) were observed in the striatum between genotypes (Figure 4C). Finally, no differences were found in the basal phosphorylation state of key components regulating the translational machinery including signaling pathways (mTORC1 and ERK), as well as translational initiation (eIF4E, eIF4G and eIF2α) and elongation (eEF2) factors (Figures 4D–F). Altogether, these results suggest that global striatal mRNA translation is not impaired in rpS6 phosphorylation deficient mice.

### Impaired Translation of a Subset of mRNAs in the Acb of rpS6^P−/−^ Mice

**Figure 5:**
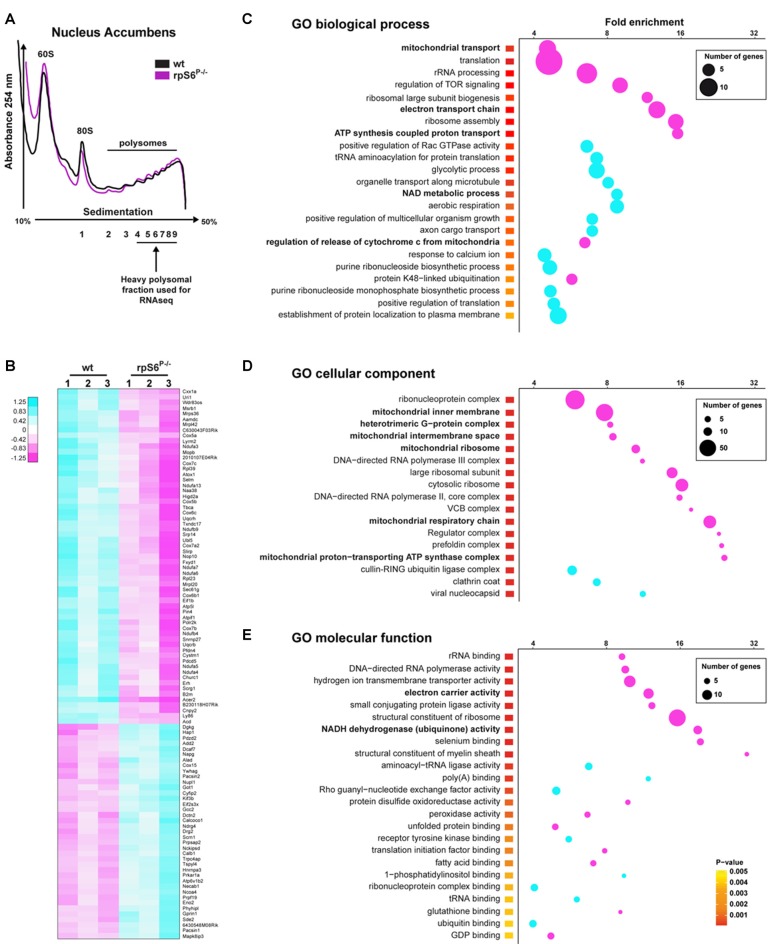
Altered translation status of a subset of mRNAs in the Acb of rpS6^P−/−^ mice. **(A)** Representative polysome profile of Acb lysates from wt (black line) and rpS6^P−/−^ (magenta line) mice (top) and schematic representation of the polysomal fractions analyzed by RNAseq (bottom). **(B)** Heatmap of the top 100 genes most significantly dysregulated by RNAseq between Acb polysomal fractions of rpS6^P−/−^ mice and wt littermates (3 replicates/genotype, 6 mice/replicate). Scaled expression values are color-coded according to the legend. **(C–E)** Enriched terms of Gene Ontology (GO) analyses including biological process **(C)**, cellular component **(D)**, and molecular function **(E)** of mRNAs whose expression is significantly changed between wt and rpS6^P−/−^ mice in polysomal fractions from Acb extracts. The fold-enrichment (cutoff > 4) is displayed for each GO terms using Fisher’s exact test (see “Materials and Methods” section). The size of the dots is proportional to the number of genes associated with a given GO term.

Despite the normal overall protein synthesis in rpS6^P−/−^ mice, we sought to assess whether basal rpS6 phosphorylation could regulate the translation of a specific subset of mRNAs, using the striatum as a test case. To address this issue, we performed polysome profiling of Acb and DS lysates followed by high-throughput RNAseq of heavy polysomal fractions, which contain the actively translated mRNAs (Figure 5A). Additionally, total mRNA abundance, reflecting the cytosolic steady-state mRNA levels, was also analyzed in whole Acb and DS lysates from both genotypes. We initially compared the fold changes of normalized read counts between genotypes in heavy polysomal fractions and whole lysates (containing cytosolic steady-state mRNAs). In the Acb, although wild-type and rpS6^P−/−^ mice displayed similar polysome profiles (Figure 5A), we identified 998 differentially expressed mRNAs, of which 497 were downregulated whereas 501 were upregulated in rpS6^P−/−^ mice. A heatmap depicting the top 100 differentially expressed genes between genotypes is shown in Figure 5B. Importantly, no changes in the normalized read counts of these 998 genes were observed in whole Acb lysates from rpS6^P−/−^ compared to wild-type littermates indicating that the described alterations did not result from changes in transcription or mRNA stability. Indeed, *Ecscr* was the only mRNA differentially modulated between the two genotypes. In contrast to the Acb, only 20 genes (*Cwc22, Myl4, Mt2, Frrs1l, Ndufa3, Sncb, Usmg5, Atp5e, Bex2, Mt1, Ndufa1, Tomm7, Nap1l5, Pcp4, 2010107E04Rik, Pebp1, Tmsb10, Sat1, Selt, Timm8b*) were differentially expressed in heavy polysomal fractions of DS lysates between genotypes, suggesting that the impact of phospho-rpS6 deficiency might be brain region-specific. To gain insight into the classes of genes differentially expressed in heavy polysomal fractions from Acb between genotypes, we performed GO analysis. Among the three domains covered by the ontology (biological process, cellular component and molecular function), terms related to mitochondrial functions are some of the most heightened, including “mitochondrial transport”, “electron transport chain”, “ATP synthesis coupled proton transport” (Figure 5C), “mitochondrial inner membrane”, “mitochondrial ribosome”, “mitochondrial respiratory chain” (Figure 5D), “electron carrier activity” and “NADH dehydrogenase (ubiquinone) activity” terms (Figure 5E), among others. Interestingly, all these terms are significantly de-enriched in rpS6^P−/−^ mice.

**Figure 6:**
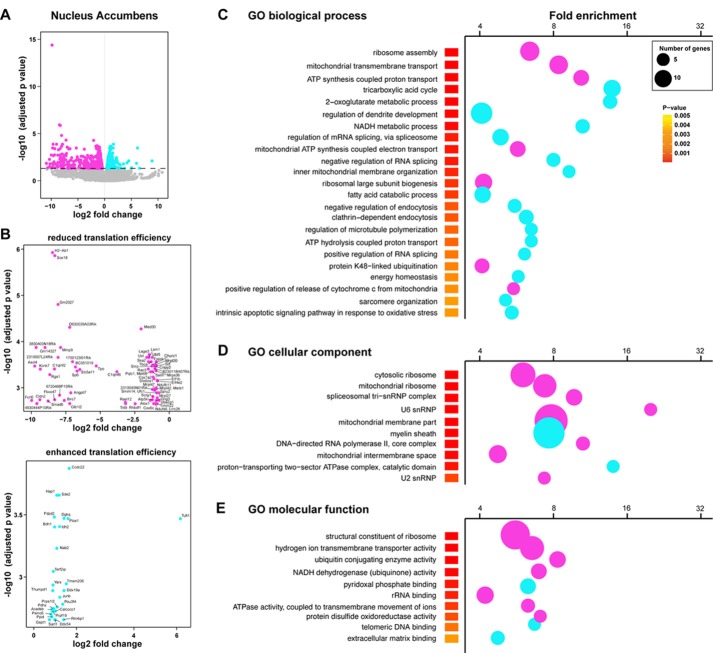
Altered translational efficiency of a subset of mRNAs in the Acb of rpS6^P−/−^ mice. **(A)** Volcano plot obtained from Xtail analysis of translational efficiency in the Acb. Log2 of the translational fold change is shown on the horizontal axis, and −log10 of the *P* value is shown on the vertical axis. Genes with significant increase or decrease in translational efficiency are represented in cyan or magenta, respectively. **(B)** Volcano plot obtained from Xtail analysis of genes with decreased (top) or increased (bottom) translational efficiency in the Acb of rpS6^P−/−^ mice **(C–E)** Enriched terms of GO analyses including biological process **(C)**, cellular component **(D)**, and molecular function **(E)** of mRNAs whose translational efficiency is significantly changed in the Acb between wt and rpS6^P−/−^. The fold-enrichment (cutoff > 4) is displayed for each GO terms using Fisher’s exact test (see “Materials and Methods” section). The size of the dots is proportional to the number of genes associated with a given GO term.

We next complemented our analysis with an additional method tool (Xtail pipeline, Xiao et al., 2016) allowing us to assess differential translation efficiency of each given mRNA on top of the transcriptional changes between genotypes. More precisely, a ratio of normalized gene counts from the RNAseq analysis on the “heavy polysomal fractions” and “whole lysates” was calculated for each gene. In line with our previous analysis, we identified 1484 genes with a statistically significant alteration in translational efficiency in the Acb (865 genes showing a decreased translational efficiency in rpS6^P−/−^ mice and 619 showing an increased translational efficiency; Figures 6A,B). In contrast, only 74 genes showed an altered heavy polysomes/whole lysate ratio between genotypes in the DS (39 genes showing a decreased translational efficiency in rpS6^P−/−^ mice and 35 showing an increased translational efficiency; Supplementary Figures S7A–C). We found a substantial overlap in the genes identified by our two analyses (Supplementary Figures S8A,B). In line with this observation, genes displaying differences in their translation efficiency belonged to similar GO terms when compared to those identified in our previous analysis (Figures 6C–E).

**Figure 7:**
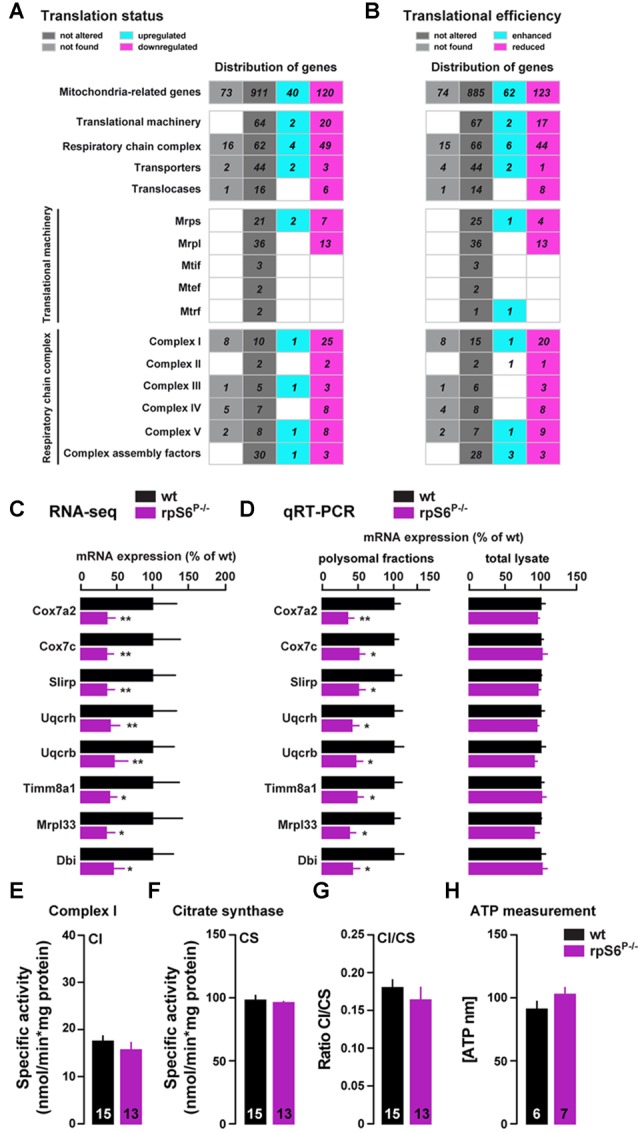
Altered translation of mitochondria-related mRNAs in rpS6^P−/−^ mice. **(A,B)** Hierarchical classification of mitochondria-related genes with altered translational status **(A)** or translational efficiency **(B)** according to MitoCarta2.0 database. The total number of genes is displayed as upregulated (green gray) or downregulated (magenta) in rpS6^P−/−^ mice, equally expressed between genotypes (dark gray), or not expressed in the Acb (light gray). **(C)** Relative mRNA abundance obtained from RNAseq of Acb heavy polysomal fractions between wt (black bars) and rpS6^P−/−^ mice (magenta bars). **(D)** Relative mRNA expression obtained by qRT-PCR analysis of heavy polysomal fractions (left) or total lysates (right) of the Acb from wt and rpS6^P−/−^ mice. The translation efficiency (polysomal association) or abundance of each mRNA was normalized to that of β-actin mRNA. Results are represented as mean ± SEM. *p < 0.05; **p < 0.01 by DESeq2 (for RNAseq) and by Student’s *t* test (for qRT-PCR; *n* and *p* values in Supplementary Table S1:7c,d). **(E)** Enzymatic activity of complex I (CI) in the Acb of wt and rpS6^P−/−^ mice. **(F)** Enzymatic activity of citrate synthase (CS) in the Acb of wt and rpS6^P−/−^ mice. **(G)** Ratio CI/CS in the Acb of wt and rpS6^P−/−^ mice. **(H)** ATP levels in the Acb of wt and rpS6^P−/−^ mice. The number of animals in each condition is indicated in the bars. Results are represented as mean ± SEM (*n* and *p* values in Supplementary Table S1:7e–h).

To further analyze the mitochondria-related genes dysregulation, we performed a hierarchical classification using the MitoCarta 2.0 inventory (Calvo et al., 2016). Indeed, among the 1071 mitochondria-related genes identified by RNAseq, 160 genes were differentially regulated in the heavy polysomal fraction between genotypes, of which 75% were downregulated and 25% upregulated in rpS6^P−/−^ mice (Figure 7A). A similar classification performed following Xtail analysis revealed 185 genes with altered translational efficiency, of which 62 and 123 have an enhanced and reduced translational efficiency, respectively (Figure 7B). Representative mitochondria-related genes found to be downregulated in Acb heavy polysomal fractions of rpS6^P−/−^ mice by RNAseq (Figure 7C) were validated by qRT-PCR analysis (Figure 7D). No changes at the transcriptional level of these genes were found in total mRNA lysates (Figure 7D) in agreement with RNAseq analysis.

Interestingly, we found that the majority of downregulated genes in knockin mice are part of the mitochondrial translational machinery (7 and 13 genes out of the 30 and 49 genes of the small and large mitochondrial ribosomal subunits, respectively; Figure 7A). On the other hand, this classification also revealed intriguing differences in genes belonging to the respiratory chain complex, since 49 genes (out of the 115 genes analyzed) were downregulated in rpS6^P−/−^ mice whereas only four were upregulated (Figure 7A). Although this bias was present in the five respiratory chain complexes, the effect was particularly evident for complex I (CI), in which 25 out of the 36 genes sequenced were downregulated in the knockin while only one was upregulated (Figure 7A).

Therefore, we next sought to analyze CI activity on total homogenates from the Acb of rpS6^P−/−^ mice and wild-type littermates. Despite the large number of downregulated genes encoding for CI proteins found in rpS6 phosphorylation-deficient mice, no alterations were observed in CI activity (Figure 7E), even after correction with the activity of CS (Figure 7F), a validated biomarker for mitochondrial density, as shown by the CI/CS ratio (Figure 7G). On the other hand, we also measured ATP levels since half of the complex V-related genes analyzed were downregulated in rpS6^P−/−^ mice. However, no differences in Acb ATP content were found between genotypes (Figure 7H). Together, our data indicate an altered translation, but not transcription, of a subset of mitochondria-related transcripts selectively in the Acb of rpS6^P−/−^ mice.

## Discussion

We describe that rpS6 phosphorylation-deficiency has an impact on Acb function in mice, affecting the translation of a subset of mRNAs, altering synaptic plasticity, and eventually causing behavioral changes. The generation of rpS6^P−/−^ knockin mice constituted a critical step forward to address the functional role of rpS6 phosphorylation in peripheral tissues (Ruvinsky et al., 2005). Studies using these mice showed an impaired cell size of mouse embryo fibroblasts, pancreatic β cells (Ruvinsky et al., 2005; Granot et al., 2009), and muscle myotubes (Ruvinsky et al., 2009), which were associated with hypoinsulinemia/glucose intolerance (Ruvinsky et al., 2005) and muscle weakness (Ruvinsky et al., 2009) respectively. However, our study shows neither brain alterations in size and weight nor neuronal morphological abnormalities, suggesting that rpS6 phosphorylation is not related to the size or morphology of neurons.

In the brain, the phosphorylation state of rpS6 has long been correlated with global and/or TOP mRNA translation after changes in synaptic plasticity (Kelleher et al., 2004; Klann and Dever, 2004; Antion et al., 2008a,b), a pharmacological stimulus (Bowling et al., 2014), or in genetic mouse models (Ricciardi et al., 2011; Bhattacharya et al., 2012). However, none of these studies established causality between rpS6 phosphorylation and protein synthesis. Our data suggests that these two phenomena are uncoupled in the brain in agreement with recent studies showing no changes in overall striatal mRNA translation despite a robust increased rpS6 phosphorylation induced by d-amphetamine, haloperidol, or papaverine administration (Biever et al., 2015a) or in the hippocampus following LTP induction (Pirbhoy et al., 2016). Here we show that steady-state ribosomes engaged in translation and *de novo* protein synthesis were unchanged in rpS6 phosphorylation-deficient mice. Although GO analyses of differential mRNAs loaded onto heavy polysomal fractions between genotypes revealed a diminution in the translation of some mRNAs (see Figures 5, 6) in rpS6^P−/−^ mice, this effect does not seem to alter global protein synthesis. The similar steady-state mRNA translation between genotypes in various brain areas (DS, Acb, hippocampus and frontal cortex) indicates that, as previously reported in non-neuronal cell types (Ruvinsky et al., 2005; Chauvin et al., 2014), rpS6 phosphorylation is dispensable for overall protein synthesis. Despite the lack of involvement of phospho-rpS6 on global mRNA translation, polysomal fractions of refed livers from rpS6^P−/−^ mice displayed a reduction in ribosome biogenesis (RiBi) transcriptional program, which includes genes involved in rRNA synthesis, cleavage, assembly with ribosomal proteins, and transport (Chauvin et al., 2014), suggesting that phospho-rpS6 controls the synthesis of RiBi factors. Our study supports this previous observation since alterations of some mRNAs involved in RiBi were observed in heavy polysomal fractions of mutant mice.

Our RNAseq analysis of Acb heavy polysomal fractions uncovered a downregulation of mitochondria-related genes in rpS6^P−/−^ mice suggesting a possible role of basal rpS6 phosphorylation in the regulation of mitochondria homeostasis. Interestingly, a decrease of three mitochondrial proteins was previously found by high-throughput proteomic analysis in the soleus of mutant mice compared to wild-type (Ruvinsky et al., 2009). The low overlap between mitochondria-related genes of the brain and muscle could be explained by the fact that only 1/3 of all mitochondrial proteins were found to be present across 14 mouse tissues analyzed (Pagliarini et al., 2008). Albeit GO analyses revealed a substantial diminution of mitochondria-related genes, our results showed indistinguishable CI and CS activities in Acb homogenates in agreement with the similar effects reported in muscles from both genotypes (Ruvinsky et al., 2009). Although in the latter study a slight alteration in ATP content was reported in the hind limb of rpS6^P−/−^ mice, we found similar ATP levels in Acb extracts from both genotypes. Conceivably, the intact mitochondria physiology observed in the Acb of rpS6^P−/−^ mice despite decreased translational efficiency of mitochondrial mRNAs could be due to compensatory mechanisms since the mutant line is not temporally inducible. On the other hand, given that the altered mRNA translation was observed only in the Acb, a putative alteration in CI activity could be masked by the presence of mitochondria in axons targeting the Acb. Moreover, while basal level mitochondrial function was unchanged, this does not rule out the possibility that under circumstances with high energy demand CI activity might be decreased in rpS6^P−/−^ mice.

Mitochondria play a pivotal role in synaptic plasticity, largely via ATP production and Ca^2+^ regulation (Mattson et al., 2008; Jeanneteau and Arango-Lievano, 2016). For example, affecting mitochondrial function using cyclosporin A (Levy et al., 2003) or rotenone (Kimura et al., 2012) results in impaired HFS-induced LTP. In this line, our study revealed an altered translational efficiency of 185 mitochondria-related genes in the Acb and a correlated HFS-induced LTP impairment in the same brain subregion. By contrast, none of these effects were observed in the DS, indicating that the impact of rpS6 phospho-deficiency is region-specific. This difference could be accounted by increased rpS6 RNA levels in the Acb compared to the DS found after sequencing the total RNA from cytosolic fractions (fold change: 1.41, *p* < 0.001). Therefore, the lack of phosphorylation might have a stronger impact in the Acb than in the DS given the higher rpS6 levels. Future studies are required to assess a causality link between altered translation of mitochondria-related mRNAs and Acb LTP alterations. Another possibility includes that the impaired HFS-induced LTP could be underlined by alterations in some genes encoding for proteins involved in receptor localization, trafficking, scaffolding, turnover, etc., as the *Rab31*, *Tmed2*, *Bcl2l1*, *Hap1*, *Mpp5*, *Pdzd2*, *Pacsin1*, *Pip5k1a*, *Sptbn4*, *Golga4*, *Acsl3*, *Pdpk1* and *Clip3* genes found by RNAseq analysis.

Pharmacological inhibition of mitochondria has been reported to cause alterations in motor behavior (Borlongan et al., 1995; Akopian et al., 2012). In our study, we found a specific alteration on novelty-induced locomotion, a behavior that requires the functional integrity of the VTA-Acb-pallidal circuit, but does not involve other basal ganglia areas such as the DS (Hooks and Kalivas, 1995). Therefore, it is tempting to speculate that the altered Acb synaptic plasticity observed in rpS6^P−/−^ mice suggests a dysfunction of the Acb, which could result in an enhanced locomotor activity only when mice are exposed to a novel environment.

Altogether, these data show that rpS6 phospho-deficiency affects the translation efficiency of a subset of mRNAs despite its dispensable role in global protein synthesis. It remains to be addressed, however, if this effect implies rpS6 to be a structural component of the ribosome or if extraribosomal functions, such as interaction with other proteins, could take place as previously reported (for review, see Biever et al., 2015b). Our study also reports an impaired Acb synaptic plasticity as well as alterations in a specific Acb-dependent behavior. Although other studies are required to assess the role of phospho-rpS6 in other brain areas, we demonstrated that this post-translational modification plays key physiological roles in the central nervous system.

## Author Contributions

EP, AB and EV conceived and led the project. EP and AB performed biochemical, histological and behavioral experiments. LC performed biochemical experiments. VP and CL conceived and performed electrophysiological recordings. SM and GM performed mitochondrial assays. JB-V performed qRT-PCR. DS led RNAseq and SR and MP performed bioinformatic analyses. OM provided rpS6 knockin mice. EP, AB and EV wrote the manuscript with input from all authors.

## Conflict of Interest Statement

The authors declare that the research was conducted in the absence of any commercial or financial relationships that could be construed as a potential conflict of interest.
