# Supplementary material for: Ribosomal Protein S6 Phosphorylation Is Involved in Novelty-Induced Locomotion, Synaptic Plasticity and mRNA Translation
Source: Front Mol Neurosci. 2017 Dec 21;10:419. doi: 10.3389/fnmol.2017.00419 (PMC5742586; doi:10.3389/fnmol.2017.00419)
Supplement: Supplementary file 1 [file Presentation_1.pdf]

Supplemental Materials

Ribosomal protein S6 phosphorylation is involved in novelty-induced locomotion, synaptic plasticity and mRNA translation

Emma Puighermanal[1]*, Anne Biever[1]*, Vincent Pascoli[2], Su Melser[3], Marine Pratlong[4], Laura Cutando[1], Stephanie Rialle[4], Dany Severac[4], Jihane Boubaker-Vitre[1], Oded Meyuhas[5], Giovanni Marsicano[3], Christian Lüscher[2,6], Emmanuel Valjent[1].

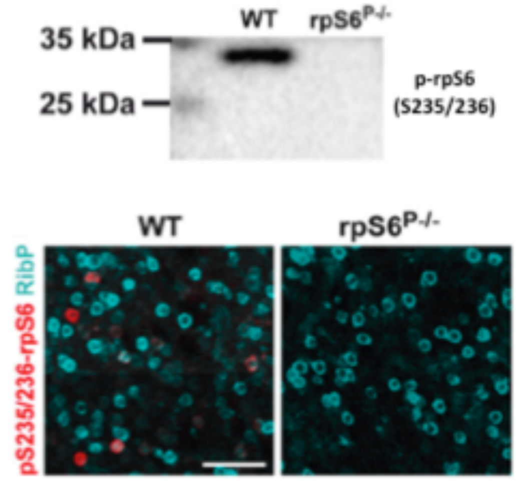

Supplementary Figure S1: Characterization of rpS6$^{P-/-}$ mice. Representative western blots of pS235/236-rpS6 in total striatal lysates from wt and rpS6$^{P-/-}$ mice (upper panel) and double immunostaining for pS235/236-rpS6 (red) and RibP (cyan) in the striatum of wt and rpS6$^{P-/-}$ mice (lower panel). Scale bar: 40 μm.

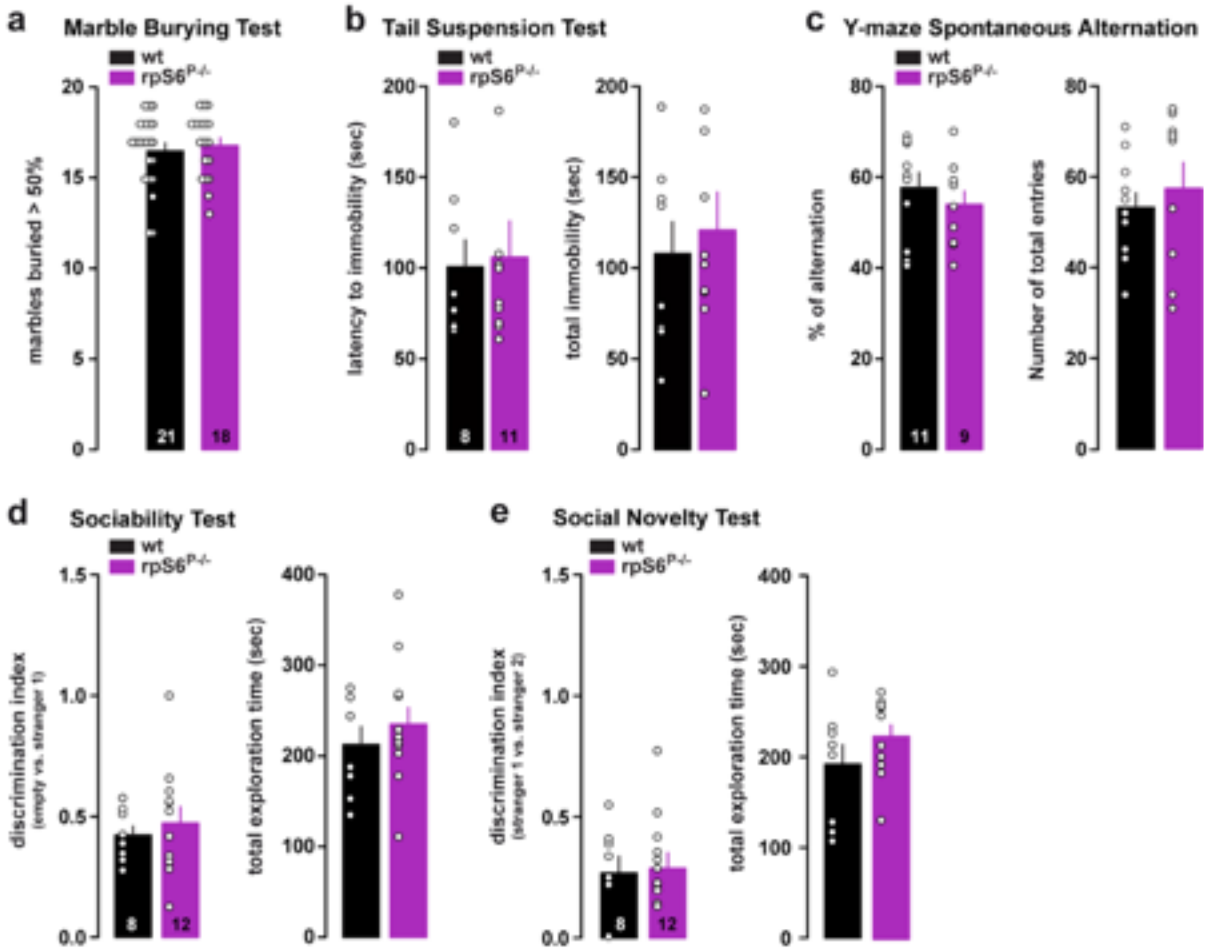

Supplementary Figure S2: Behavioral analysis of rpS6$^{P-/-}$ mice. (**a**) Marble burying test. Number of marbles buried more than 50%. (**b**) Tail suspension test. Latency to immobility (sec) (left) and total time spent immobile (sec) (right). (**c**) Y-maze spontaneous alternation test. Percentage of arm alternation (left) and number of total arm entries (right). (**d** and **e**) Three-chamber social interaction test. (**d**) Discrimination index values for the sociability test; S1, wire-cage with stranger 1 (social stimulus); empty wire-cage compartment (nonsocial stimulus) (left) and the total time spent sniffing both wire-cages (right). (**e**) Discrimination index values for the social novelty three test; S1, wire-cage with stranger 1 (previously encountered mouse); S2, wire-cage with stranger 2 (novel social stimulus). The number of animals in each condition is indicated in the bars. Results are represented as scatter plots and means ± SEM., wt (black bars) and rpS6$^{P-/-}$ (magenta bars), (n and p values in Supplementary Table 1: S2a-e).

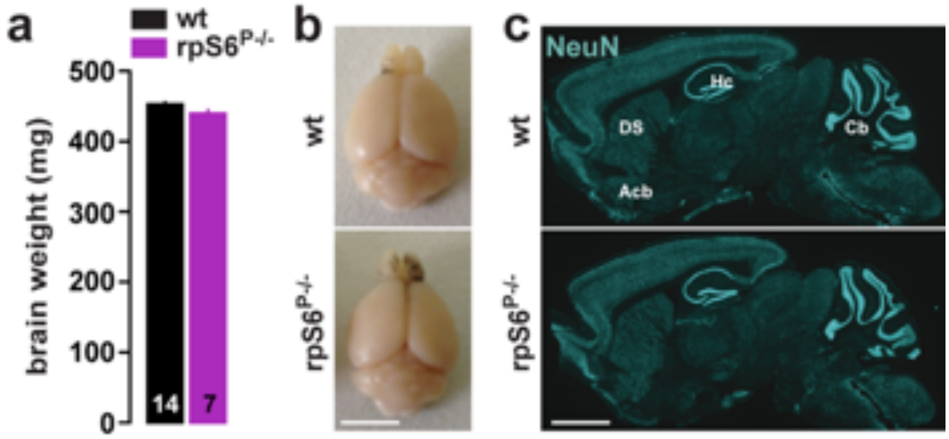

Supplementary Figure S3: Brain morphology of rpS6$^{P-/-}$ mice. (**a**) Average brain weight (mg) from wt (black bars) and rpS6$^{P-/-}$ (magenta bars) mice. The number of animals in each condition is indicated in the bars. Results are represented as means ± SEM. (n and p values in Supplementary Table 1: S3a). (**b**) Representative brain from a wt and rpS6$^{P-/-}$ mouse. Scale bar: 4.5 mm. (**c**) Immunostaining for NeuN in sagittal brain sections of wt (top) and rpS6$^{P-/-}$ (bottom) mice. Scale bar: 2.5 mm.

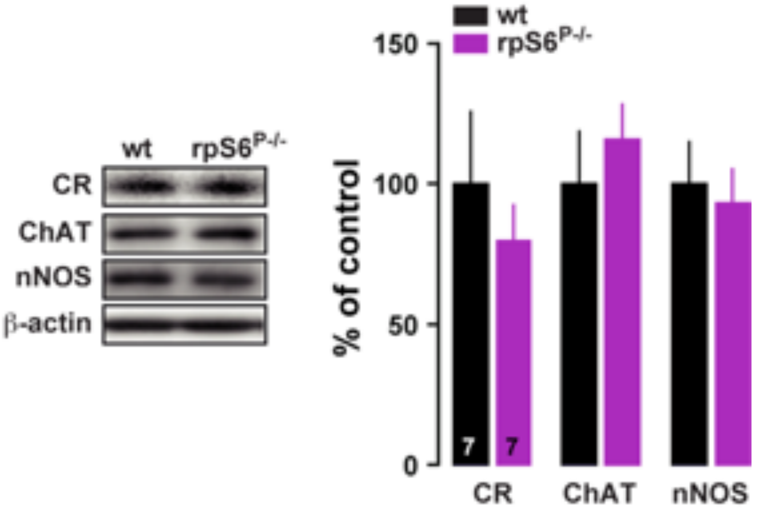

Supplementary Figure 4: Striatal expression levels of calretinin, choline acetyltransferase, and nNOS in rpS6$^{P-/-}$ mice. Representative western blots (left) quantified signals (right) of calretinin (CR), choline acetyltransferase (ChAT) and nNOS normalized to β-actin in whole striatal lysates from wt (black bars) and rpS6$^{P-/-}$ (magenta bars) mice. The number of animals in each condition is indicated in the bars. Results are represented as means ± SEM. (n and p values in Supplementary Table 1: S4).

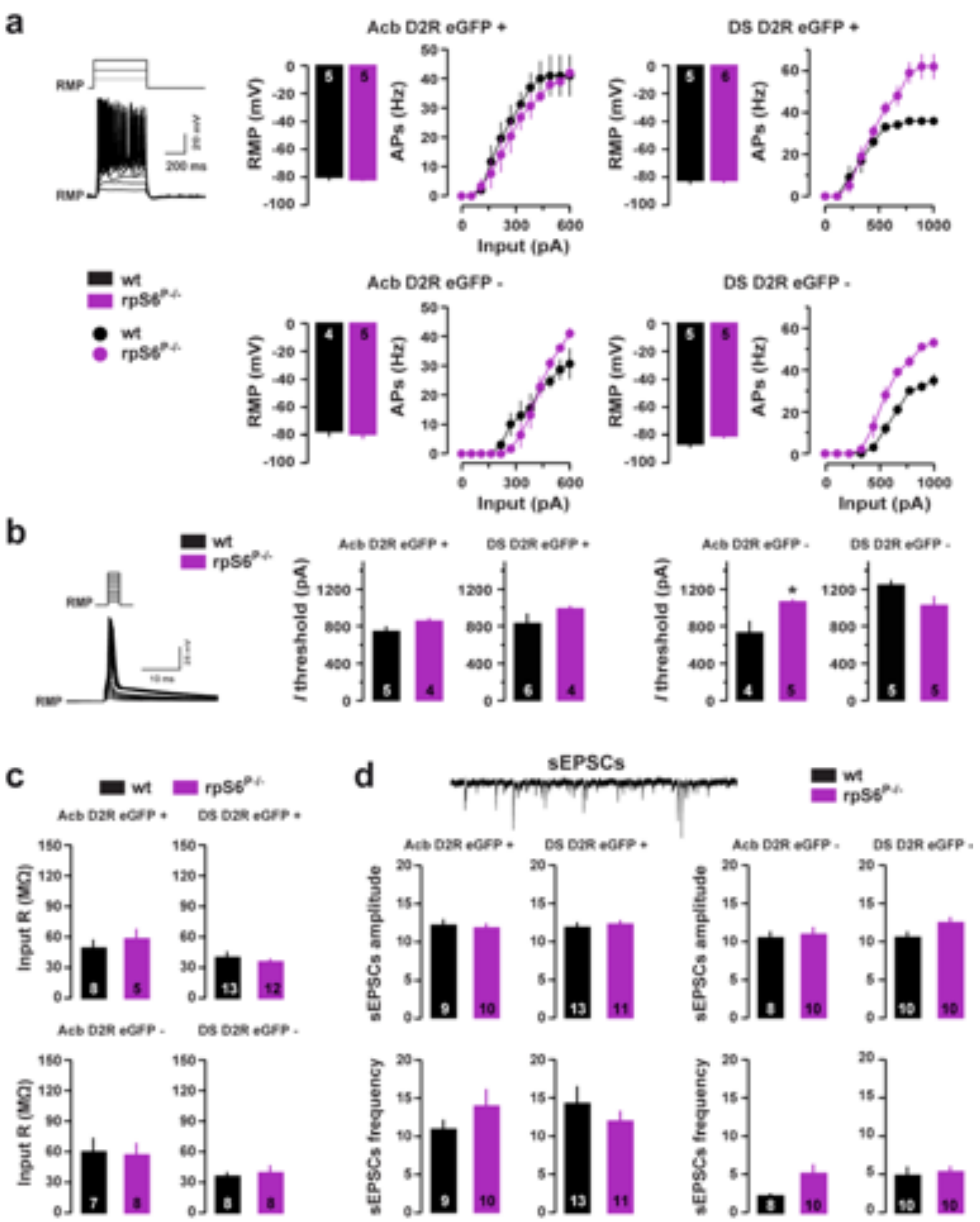

Supplementary Figure S5: Electrophysiological parameters in rpS6$^{P-/-}$ mice. (**a**) Resting membrane potential (RMP) and input-output curves (number of action potentials observed with increasing current injection) in D2-MSNs (upper panels) and GFP negative, putative D1-MSNs (lower panels) of the nucleus accumbens (Acb) and the dorsal striatum (DS). No significant difference was observed. (**b)** Action potential threshold in response to a short current injection (2.5 ms). No differences were observed, except in D2-MSNs of the Acb. (**c**) Input resistance and (**d**) spontaneous excitatory postsynaptic currents (sEPSCs) were similar between the two genotypes in both cell types in the Acb and DS reflecting similar synaptic connectivity. The number of cells in

each condition is indicated in the bars. Results are represented as means ± SEM. Statistical analysis, Student's *t* test (n and p values in Supplementary Table 1: S5a-d), * $p < 0.05$.

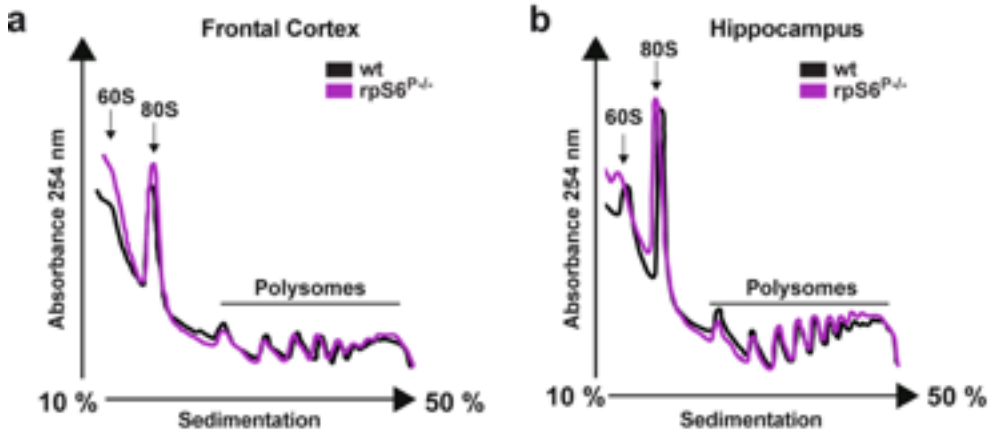

Supplementary Figure S6: Polysome profiling analysis in various brain regions of rpS6$^{P-/-}$ mice. Representative polysome profile of a lysate from the frontal cortex (**a**) and the hippocampus (**b**) of wt (black lines) and rpS6$^{P-/-}$ (magenta lines) mice (n = 3 mice/group).

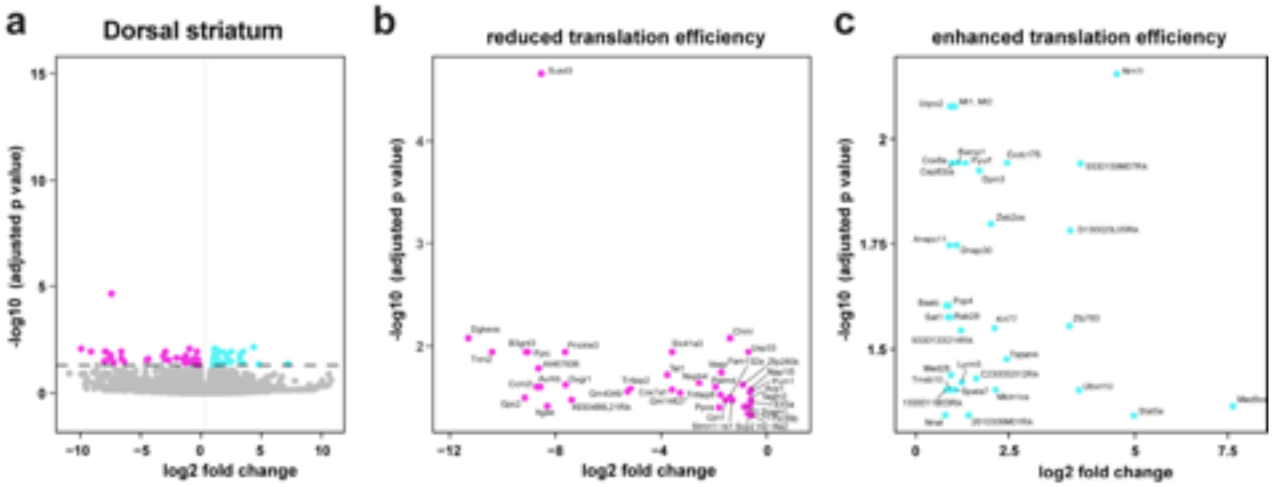

Supplementary Figure S7: Altered translational efficiency of a subset of mRNAs in the DS of rpS6$^{P-/-}$ mice. (**a**) Volcano plot obtained from Xtail analysis of translational efficiency in the DS. Log2 of the translational fold change is shown on the horizontal axis and −log10 of the *P* value is shown on the vertical axis. Genes with significant increase or decrease in translational efficiency are represented in cyan or magenta, respectively. (**b** and **c**) Volcano plot obtained from Xtail analysis of genes with decreased (**b**) or increased (**c**) translational efficiency in the DS of rpS6$^{P-/-}$ mice.

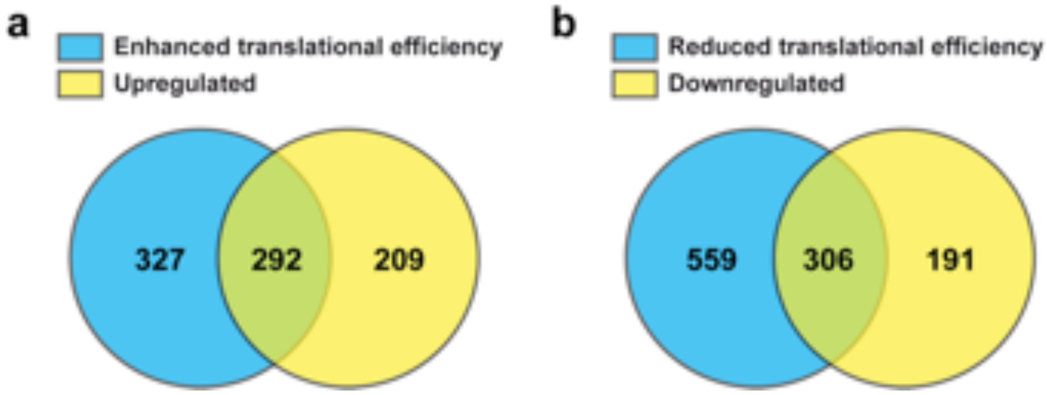

Supplementary Figure S8: Venn diagrams of genes with significantly altered translational status and/or translational efficiency in the Acb of rpS6$^{P-/-}$ mice. (**a**) Number of genes found to be upregulated in heavy polysomal fractions (yellow) or displaying an enhanced translational efficiency (blue) in rpS6$^{-/-}$ mice compared to wild-type littermates. (**b**) Number of genes found to be downregulated in heavy polysomal fractions (yellow) or displaying a reduced translational efficiency (blue) in rpS6$^{-/-}$ mice compared to wild-type littermates.

Supplementary Table 1: Statistical Analysis

**Figure**	**Groups** (n: number of mice)	**Statistical Analysis**
1a	wt (n = 15)<br>rpS6$^{P-/-}$ (n = 15)	Time-point 10 min: $t_{(28)}$ = 3.427, p = 0.0019<br>Time-point 20 min: $t_{(28)}$ = 2.027, p = 0.0522
1b	wt (n = 15)<br>rpS6$^{P-/-}$ (n = 15)	Time-point 10 min: $t_{(26)}$ = 0.3319, p = 0.7374<br>Time-point 20 min: $t_{(26)}$ = 0.9122, p = 0.3701
1d	wt (n = 12)<br>rpS6$^{P-/-}$ (n = 14)	$t_{(24)}$ = 2.359, p = 0.0268
1e	wt (n = 12)<br>rpS6$^{P-/-}$ (n = 14)	$t_{(24)}$ = 2.452, p = 0.0219
1f	wt (n = 12)<br>rpS6$^{P-/-}$ (n = 14)	$t_{(24)}$ = 2.123, p = 0.0443
1g	wt (n = 12)<br>rpS6$^{P-/-}$ (n = 14)	$t_{(24)}$ = 2.713, p = 0.0121
1h	wt (n = 12)<br>rpS6$^{P-/-}$ (n = 12)	$t_{(22)}$ = 0.2430, p = 0.8102
1i	wt (n = 10)<br>rpS6$^{P-/-}$ (n = 11)	$t_{(19)}$ = 0.04123, p = 0.9675
1j	wt (n = 10)<br>rpS6$^{P-/-}$ (n = 11)	$t_{(19)}$ = 0.8220, p = 0.4213
1k	wt (n = 10)<br>rpS6$^{P-/-}$ (n = 11)	$t_{(19)}$ = 2.189, p = 0.0413
2e	wt (n = 7)<br>rpS6$^{P-/-}$ (n = 7)	D2R: $t_{(12)}$ =1.197, p = 0.1909<br>DARPP-32: $t_{(12)}$ = 0.1254, p = 0.9023<br>DAT: $t_{(12)}$ = 1.417, p = 0.1819<br>Gαolf: $t_{(12)}$ = 0.08516, p = 0.91610<br>TH: $t_{(12)}$ = 0.04341, p = 0.9335<br>MAO-A: $t_{(12)}$ = 0.3632, p = 0.7228<br>VMAT2: $t_{(12)}$ = 0.3074, p = 0.7638<br>NET: $t_{(12)}$ = 0.6870, p = 0.5052
2f	wt (n = 6)<br>rpS6$^{P-/-}$ (n = 7)	DS: $t_{(11)}$ = 0.8443, p = 0.4165<br>Acb: $t_{(11)}$ = 1.108, p = 0.2913
2h	wt (n = 6)<br>rpS6$^{P-/-}$ (n = 7)	Thin: DS: $t_{(11)}$ = 0.3288, p = 0.7485<br>Acb: $t_{(11)}$ = 0.8555, p = 0.4105<br>Stubby: DS: $t_{(11)}$ = 0.9307, p = 0.3720<br>Acb: $t_{(11)}$ = 0.5338, p = 0.6041<br>Mushroom: DS: $t_{(11)}$ = 0.7044, p = 0.4958<br>Acb: $t_{(11)}$ = 1.179, p = 0.2634
2i	DS<br>wt (n = 15)	DS<br>Syp: $t_{(30)}$ = 1.330, p = 0.1936

	$rpS6^{P-/-}$ (n = 16)<br><br>Acb<br>wt (n = 15)<br>$rpS6^{P-/-}$ (n = 17)	PSD95: $t_{(30)}$ = 0.9467, p = 0.3514<br><br>Acb<br>Syp: $t_{(29)}$ = 0.3161, p = 0.7542<br>PSD95: $t_{(29)}$ = 0.5202, p = 0.6069
3a	wt (n = 8)<br>$rpS6^{P-/-}$ (n = 5)	$t_{(11)}$ = 5.685, p = 0.0001
3b	wt (n = 12)<br>$rpS6^{P-/-}$ (n = 12)	$t_{(22)}$ = 0.6017, p = 0.5536
3c	wt (n = 7)<br>$rpS6^{P-/-}$ (n = 8)	$t_{(13)}$ = 2.698, p = 0.0183
3d	wt (n = 8)<br>$rpS6^{P-/-}$ (n = 8)	$t_{(14)}$ = 1.368, p = 0.1938
4b	wt (n = 7)<br>$rpS6^{P-/-}$ (n = 7)	$t_{(12)}$ = 0.5424, p = 0.5974
4c	wt (n = 14)<br>$rpS6^{P-/-}$ (n = 12)	rpS6: $t_{(24)}$ = 0.1182, p = 0.9069<br>eEF1A: $t_{(24)}$ = 0.5571, p = 0.5826
4d	wt (n = 20)<br>$rpS6^{P-/-}$ (n = 22)	pS2448-mTOR: $t_{(40)}$ = 0.5962, p = 0.5544<br>pS1108-eIF4G: $t_{(40)}$ = 0.5378, p = 0.5937<br>pT389-p70S6K: $t_{(40)}$ = 0.2966, p = 0.7683<br>pT37/46-4EBP1: $t_{(40)}$ =0.6754, p = 0.5033
4e	wt (n = 18)<br>$rpS6^{P-/-}$ (n = 16)	pS51-eIF2α: $t_{(32)}$ = 0.09879, p = 0.9219<br>pT56-eEF2: $t_{(32)}$ = 0.3839, p = 0.7036
4f	wt (n = 11)<br>$rpS6^{P-/-}$ (n = 11)	pT185/Y187-ERK1/2: $t_{(20)}$ = 0.09145, p = 0.9280<br>pS209-eIF4E: $t_{(20)}$ = 0.3014, p = 0.7662<br>pT421/S424-p70S6K: $t_{(20)}$ = 1.593, p = 0.1268
7c	wt (3 replicates, n = 2 per replicate)<br>$rpS6^{P-/-}$ (3 replicates, n = 2 per replicate)	DESeq2<br>Cox7a2 padj = 0.002130559<br>Cox7c padj = 0.005196321<br>Slirp padj = 0.002914695<br>Uqcrh padj = 0.005196321<br>Uqcrb padj = 0.008156897<br>Timm8a1 padj = 0.013141037<br>Mrpl33 padj = 0.013747103<br>Dbi padj = 0.014858228
7d, left	wt (3 replicates, n = 2 per replicate)<br>$rpS6^{P-/-}$ (3 replicates, n = 2 per replicate)	Cox7a2: $t_{(4)}$ = 5.499, p = 0.0053<br>Cox7c: $t_{(4)}$ = 4.328, p = 0.0124<br>Slirp: $t_{(4)}$ = 3.299, p = 0.03<br>Uqcrh: $t_{(4)}$ = 3.690, p = 0.0210<br>Uqcrb: $t_{(4)}$ = 3.094, p = 0.0364<br>Timm8a1: $t_{(4)}$ = 3.426, p = 0.0266<br>Mrpl33: $t_{(4)}$ = 4.552, p = 0.0104

		Dbi: $t_{(4)}$ = 3.151, p = 0.0345
7d, right	wt (3 replicates, n = 2 per replicate)<br>rpS6$^{P-/-}$ (3 replicates, n = 2 per replicate)	Cox7a2: $t_{(4)}$ = 0.9086, p = 0.4149<br>Cox7c: $t_{(4)}$ = 0.05755, p = 0.9569<br>Slirp: $t_{(4)}$ = 1.044, p = 0.3555<br>Uqcrh: $t_{(4)}$ = 0.8096, p = 0.3646<br>Uqcrb: $t_{(4)}$ = 1.195, p = 0.2980<br>Timm8a1: $t_{(4)}$ = 0.02178, p = 0.9837<br>Mrpl33: $t_{(4)}$ = 1.196, p = 0.2979<br>Dbi: $t_{(4)}$ = 0.08686, p = 0.9350
7e	wt (n = 15)<br>rpS6$^{P-/-}$ (n = 13)	$t_{(26)}$ = 0.923, p = 0.3646
7f	wt (n = 15)<br>rpS6$^{P-/-}$ (n = 13)	$t_{(26)}$ = 0.4254, p = 0.6740
7g	wt (n = 15)<br>rpS6$^{P-/-}$ (n = 13)	$t_{(26)}$ = 0.7890, p = 0.4373
7h	wt (n = 6)<br>rpS6$^{P-/-}$ (n = 7)	$t_{(11)}$ =1.315, p = 0.2154
S2a	wt (n = 21)<br>rpS6$^{P-/-}$ (n = 18)	$t_{(37)}$ = 0.4856, p = 0.6301
S2b	wt (n = 8)<br>rpS6$^{P-/-}$ (n = 11)	Latency to immobility: $t_{(17)}$ = 0.2248, p = 0.8248<br>Total immobility: $t_{(17)}$ = 0.6868, p = 0.3971
S2c	wt (n = 11)<br>rpS6$^{P-/-}$ (n = 9)	% of alternation $t_{(18)}$ = 0.8440, p = 0.4097<br>Total arm entries $t_{(18)}$ = 0.6669, p = 0.5133
S2d	wt (n = 8)<br>rpS6$^{P-/-}$ (n = 12)	DI: $t_{(18)}$ = 0.6005, p = 0.5557<br>Total exploration time: $t_{(18)}$ = 0.7625, p = 0.4556
S2e	wt (n = 8)<br>rpS6$^{P-/-}$ (n = 12)	DI: $t_{(18)}$ = 0.2196, p = 0.8287<br>Total exploration time: $t_{(18)}$ = 1.359, p = 0.1901
S3a	wt (n = 14)<br>rpS6$^{P-/-}$ (n = 7)	$t_{(19)}$ = 1.703, p = 0.1050
S4	wt (n = 7)<br>rpS6$^{P-/-}$ (n = 7)	CR: $t_{(12)}$ = 0.6970, p = 0.4991<br>ChAT: $t_{(12)}$ = 0.6627, p = 0.5200<br>nNOS: $t_{(12)}$ = 0.3397, p = 0.7400
S5a	RMP<br>wt (n = 5); rpS6$^{P-/-}$ (n = 5)<br>wt (n = 5); rpS6$^{P-/-}$ (n = 6)<br>wt (n = 4); rpS6$^{P-/-}$ (n = 5)<br>wt (n = 5); rpS6$^{P-/-}$ (n = 5)	RMP<br>Acb D2R eGFP$^+$: $t_{(8)}$ = 0.7115, p = 0.4970<br>DS D2R eGFP$^+$: $t_{(9)}$ = 1.145, p = 0.2817<br>Acb D2R eGFP$^-$: $t_{(7)}$ = 0.9585, p = 0.3697<br>DS D2R eGFP$^-$: $t_{(8)}$ = 1.041, p = 0.3283
S5b	*I* Threshold<br>wt (n = 5); rpS6$^{P-/-}$ (n = 4)<br>wt (n = 6); rpS6$^{P-/-}$ (n = 4)<br>wt (n = 4); rpS6$^{P-/-}$ (n = 5)<br>wt (n = 5); rpS6$^{P-/-}$ (n = 5)	*I* Threshold<br>Acb D2R eGFP$^+$: $t_{(7)}$ = 1.742, p = 0.1250<br>DS D2R eGFP$^+$: $t_{(8)}$ = 1.655, p = 0.1366<br>Acb D2R eGFP$^-$: $t_{(7)}$ = 2.958, p = 0.0212<br>DS D2R eGFP$^-$: $t_{(8)}$ = 1.687, p = 0.1300

S5c	Inputs<br>wt (n = 8); $rpS6^{P-/-}$ (n = 5)<br>wt (n = 13); $rpS6^{P-/-}$ (n = 12)<br>wt (n = 7); $rpS6^{P-/-}$ (n = 8)<br>wt (n = 8); $rpS6^{P-/-}$ (n = 8)	Inputs<br>Acb D2R $eGFP^{+}$: $t_{(11)} = 0.7287$, p = 0.4814<br>DS D2R $eGFP^{+}$: $t_{(23)} = 0.7211$, p = 0.4781<br>Acb D2R $eGFP^{-}$: $t_{(13)} = 0.1937$, p = 0.8494<br>DS D2R $eGFP^{-}$: $t_{(14)} = 0.4274$, p = 0.6755
S5d	Amplitude/Frequency<br>wt (n = 9); $rpS6^{P-/-}$ (n = 10)<br>wt (n = 13); $rpS6^{P-/-}$ (n = 11)<br>wt (n = 8); $rpS6^{P-/-}$ (n = 10)<br>wt (n = 10); $rpS6^{P-/-}$ (n = 10)	Amplitude<br>Acb D2R $eGFP^{+}$: $t_{(17)} = 0.3897$, p = 0.7016<br>DS D2R $eGFP^{+}$: $t_{(22)} = 0.5173$, p = 0.6101<br>Acb D2R $eGFP^{-}$: $t_{(16)} = 0.3249$, p = 0.7494<br>DS D2R $eGFP^{-}$: $t_{(18)} = 1.893$, p = 0.0746<br><br>Frequency<br>Acb D2R $eGFP^{+}$: $t_{(17)} = 1.235$, p = 0.2337<br>DS D2R $eGFP^{+}$: $t_{(22)} = 0.8479$, p = 0.4056<br>Acb D2R $eGFP^{-}$: $t_{(16)} = 2.041$, p = 0.0581<br>DS D2R $eGFP^{-}$: $t_{(14)} = 0.3272$, p = 0.7473
